# Genetic evidence suggests posttraumatic stress disorder as a subtype of major depressive disorder

**DOI:** 10.1172/JCI145942

**Published:** 2022-02-01

**Authors:** Fuquan Zhang, Shuquan Rao, Hongbao Cao, Xiangrong Zhang, Qiang Wang, Yong Xu, Jing Sun, Chun Wang, Jiu Chen, Xijia Xu, Ning Zhang, Lin Tian, Jianmin Yuan, Guoqiang Wang, Lei Cai, Mingqing Xu, Ancha Baranova

**Affiliations:** 1 Institute of Neuropsychiatry, and; 2 Department of Psychiatry, The Affiliated Brain Hospital of Nanjing Medical University, Nanjing, China.; 3 School of Life Science and Engineering, Southwest Jiao Tong University, Chengdu, China.; 4 School of Systems Biology, George Mason University, Manassas, Virginia, USA.; 5 Department of Geriatric Psychiatry, The Affiliated Brain Hospital of Nanjing Medical University, Nanjing, China.; 6 Mental Health Center and Psychiatric Laboratory, State Key Laboratory of Biotherapy, and; 7 West China Brain Research Center, West China Hospital of Sichuan University, Chengdu, China.; 8 Department of Psychiatry, First Clinical Medical College/First Hospital of Shanxi Medical University, Taiyuan, China.; 9 Institute of Brain Functional Imaging, Nanjing Medical University, Nanjing, China.; 10 Institute of Neuropsychiatry, The Affiliated Brain Hospital of Nanjing Medical University, Nanjing, China.; 11 Wuxi Mental Health Center of Nanjing Medical University, Wuxi, China.; 12 Bio-X Institutes, Key Laboratory for the Genetics of Developmental and Neuropsychiatric Disorders (Ministry of Education), and; 13 Shanghai Key Laboratory of Psychotic Disorders, Shanghai Mental Health Center, Shanghai Jiao Tong University, Shanghai, China.; 14 Research Centre for Medical Genetics, Moscow, Russia.

**Keywords:** Genetics, Depression, Genetic variation, Molecular genetics

## Abstract

**BACKGROUND:**

Major depressive disorder (MDD) and posttraumatic stress disorder (PTSD) are highly comorbid and exhibit strong correlations with one another. We aimed to investigate mechanisms of underlying relationships between PTSD and 3 kinds of depressive phenotypes, namely, MDD, depressed affect (DAF), and depression (DEP, including both MDD and the broad definition of depression).

**METHODS:**

Genetic correlations between PTSD and the depressive phenotypes were tested using linkage disequilibrium score regression. Polygenic overlap analysis was used to estimate shared and trait-specific causal variants across a pair of traits. Causal relationships between PTSD and the depressive phenotypes were investigated using Mendelian randomization. Shared genomic loci between PTSD and MDD were identified using cross-trait meta-analysis.

**RESULTS:**

Genetic correlations of PTSD with the depressive phenotypes were in the range of 0.71–0.80. The estimated numbers of causal variants were 14,565, 12,965, 10,565, and 4,986 for MDD, DEP, DAF, and PTSD, respectively. In each case, causal variants contributing to PTSD were completely or largely covered by causal variants defining each of the depressive phenotypes. Mendelian randomization analysis indicated that the genetically determined depressive phenotypes confer a causal effect on PTSD (*b* = 0.21–0.31). Notably, genetically determined PTSD confers a causal effect on DEP (*b* = 0.14) and DAF (*b* = 0.15), but not MDD. Cross-trait meta-analysis of MDD and PTSD identified 47 genomic loci, including 29 loci shared between PTSD and MDD.

**CONCLUSION:**

Evidence from shared genetics suggests that PTSD is a subtype of MDD. This study provides support to the efforts in reducing diagnostic heterogeneity in psychiatric nosology.

**FUNDING:**

The National Key Research and Development Program of China and the National Natural Science Foundation of China.

## Graphical abstract

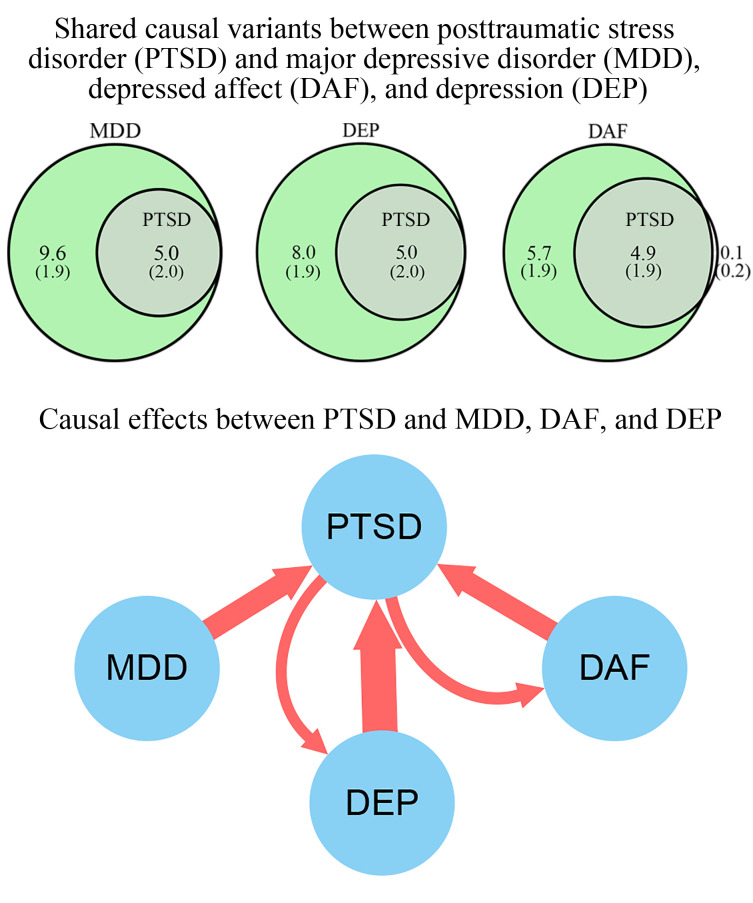

## Introduction

Mental disorders confer a heavy burden on society (1). Major depressive disorder (MDD), which manifests as a persistently low mood, is the most prevalent mental disorder worldwide and is accompanied by considerable morbidity, mortality, and high risk of suicide (2). Posttraumatic stress disorder (PTSD) is a chronic, impairing disorder characterized by intrusive trauma-related memories, hypervigilance to and avoidance of trauma-related cues, and negative emotionality. A cross-national survey revealed a lifetime prevalence of PTSD as 3.9% of the total sample, and as 5.6% of those exposed to trauma (3).

PTSD is a highly comorbid disorder. A majority of PTSD patients also meet the criteria for one or more additional psychiatric disorders, with MDD being a prevalent comorbidity (4, 5). Epidemiological studies have reported that 52% of individuals with PTSD are codiagnosed with MDD (6). Comorbidity with MDD is regarded as an obstacle to proper PTSD diagnosing (7). In its symptoms, PTSD also overlaps with other mood and anxiety disorders, including anhedonia, sleep difficulty, irritability, and difficulty in concentrating. The high levels of comorbidity and symptom overlap raise questions about the validity of the entire PTSD construct (8–11).

Although a high rate of PTSD-MDD co-occurrence has been well established, little is known about the shared pathophysiological mechanisms. Identifying the genetic underpinnings of this comorbidity is necessary for uncovering phenotypic relationships between MDD and PTSD. The genetic correlation coefficient (*r_g_*) is a prevailing measure to quantify the genetic relationship between 2 traits, with the sign of the correlation coefficient being used as an indicator for the direction of the shared genetic effects. However, when dealing with mixtures of effect directions across shared genetic variants, genetic correlation analyses may be underpowered (12). Because of that, the polygenic overlap was recently proposed to measure the fraction of genetic variants causally associated with both traits over the total number of causal variants across a pair of traits involved (12).

In this study, we estimate genetic correlation and polygenic overlap between PTSD and the depressive phenotypes and perform multi-SNP Mendelian randomization (MR) analysis on summary results presented in GWAS data sets. Next, cross-trait meta-analyses were employed to identify the pleiotropic genomic loci and the genes shared between MDD and PTSD. Finally, we discuss the potential benefits of integrating PTSD as a subtype of MDD.

## Results

### Genetic correlation and polygenic overlap analysis.

**Figure 1:**
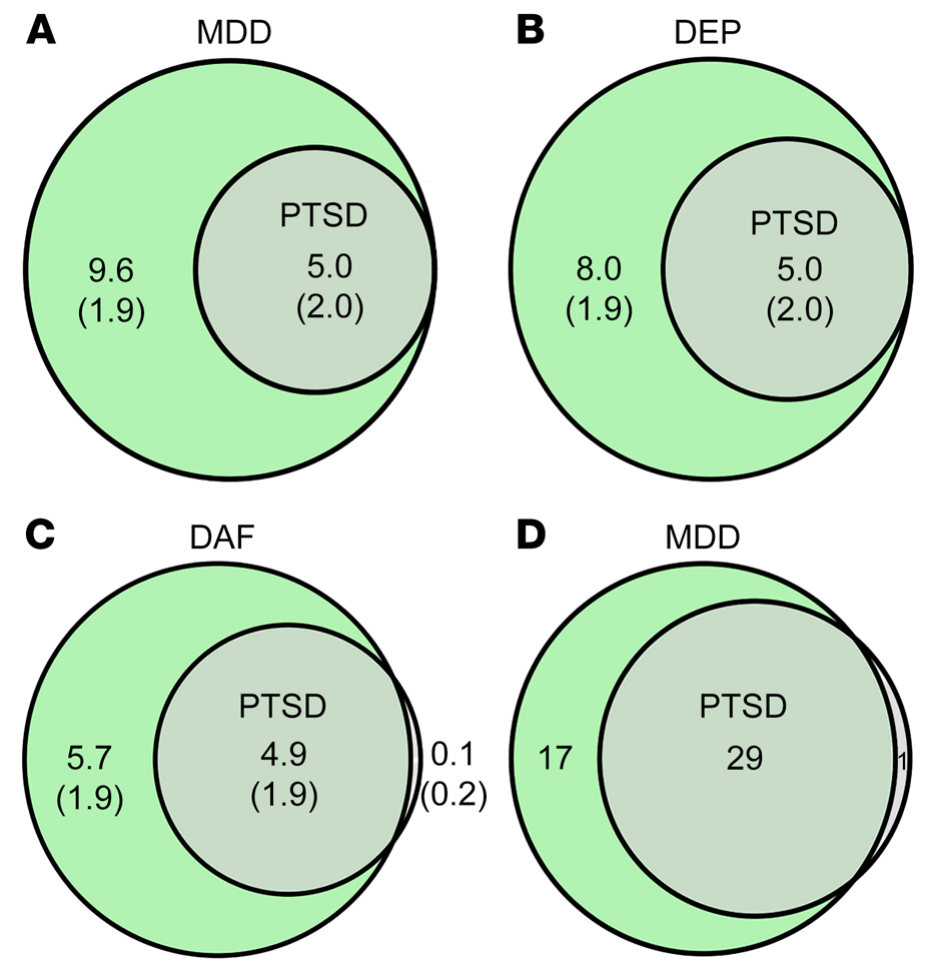
Shared causal variants and genomic loci between the depressive phenotypes and posttraumatic stress disorder (PTSD). (**A**–**C**) Venn diagrams of unique and shared causal variants, showing polygenic overlap between PTSD and the depressive phenotypes. The numbers indicate the estimated quantity of causal variants (in thousands) per component, explaining 90% of SNP-attributed heritability for each phenotype. The numbers within parentheses indicate SEM. DEP, depression; DAF, depressed affect. (**D**) Venn diagram of genomic loci that overlap between major depressive disorder (MDD) and PTSD. The numbers indicate amounts of genomic loci either unique for each condition or shared between MDD and PTSD.

As shown in Table 1, PTSD displays a high genetic correlation with the depressive phenotypes (*r_g_* = 0.71–0.80). The estimated amounts of causal variants were 14,565 ± 706, 12,965 ± 350, 10,565 ± 453, and 4,986 ± 1960 for MDD, depression (DEP), depressed affect (DAF), and PTSD, respectively. Summarily, these causal variants contribute 90% of the heritability for each trait. In each case, causal variants contributing to PTSD were completely or nearly covered by causal variants defining each of the depressive phenotypes analyzed (Figure 1, A–C).

### MR analysis.

**Figure 2:**
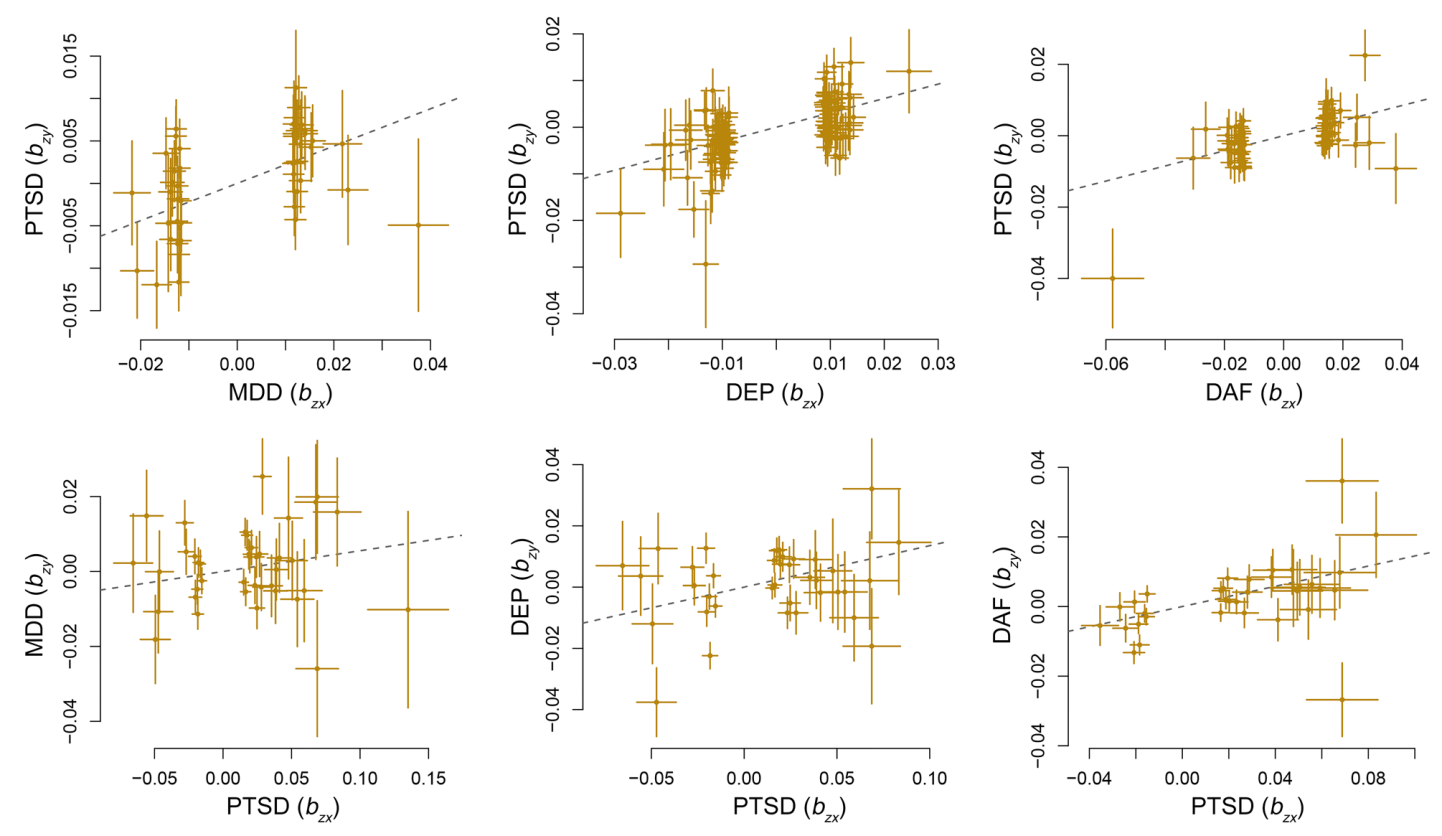
The causal effect between posttraumatic stress disorder (PTSD) and major depressive disorder (MDD), depression (DEP), and depressed affect (DAF). The trait on the *x* axis denotes exposure, the trait on the *y* axis denotes outcome, and each cross point represents an instrumental variant. The lines denote effect sizes (*b*) of exposure on outcome.

MR analysis indicates that the genetically determined depressive phenotypes confer a causal effect on PTSD (*b* = 0.21–0.31, Table 1). Notably, genetically determined PTSD confers a causal effect on DEP (*b* = 0.14) and DAF (*b* = 0.15) phenotypes, but not MDD (Table 1 and Figure 2). At a *P*-value threshold of 5 × 10^–8^, there were not enough instrumental variants to analyze the causal effect of PTSD on the depressive phenotypes; therefore, a *P*-value threshold of 1 × 10^–5^ was used. MR-Egger analysis did not support any pleiotropic effect biasing the estimates of the causal effects between PTSD and the depressive phenotypes (MR-Egger intercept < 0.01, *P* > 0.05, Supplemental Table 1; supplemental material available online with this article; https://doi.org/10.1172/JCI145942DS1).

### Cross-trait meta-analysis.

**Figure 3:**
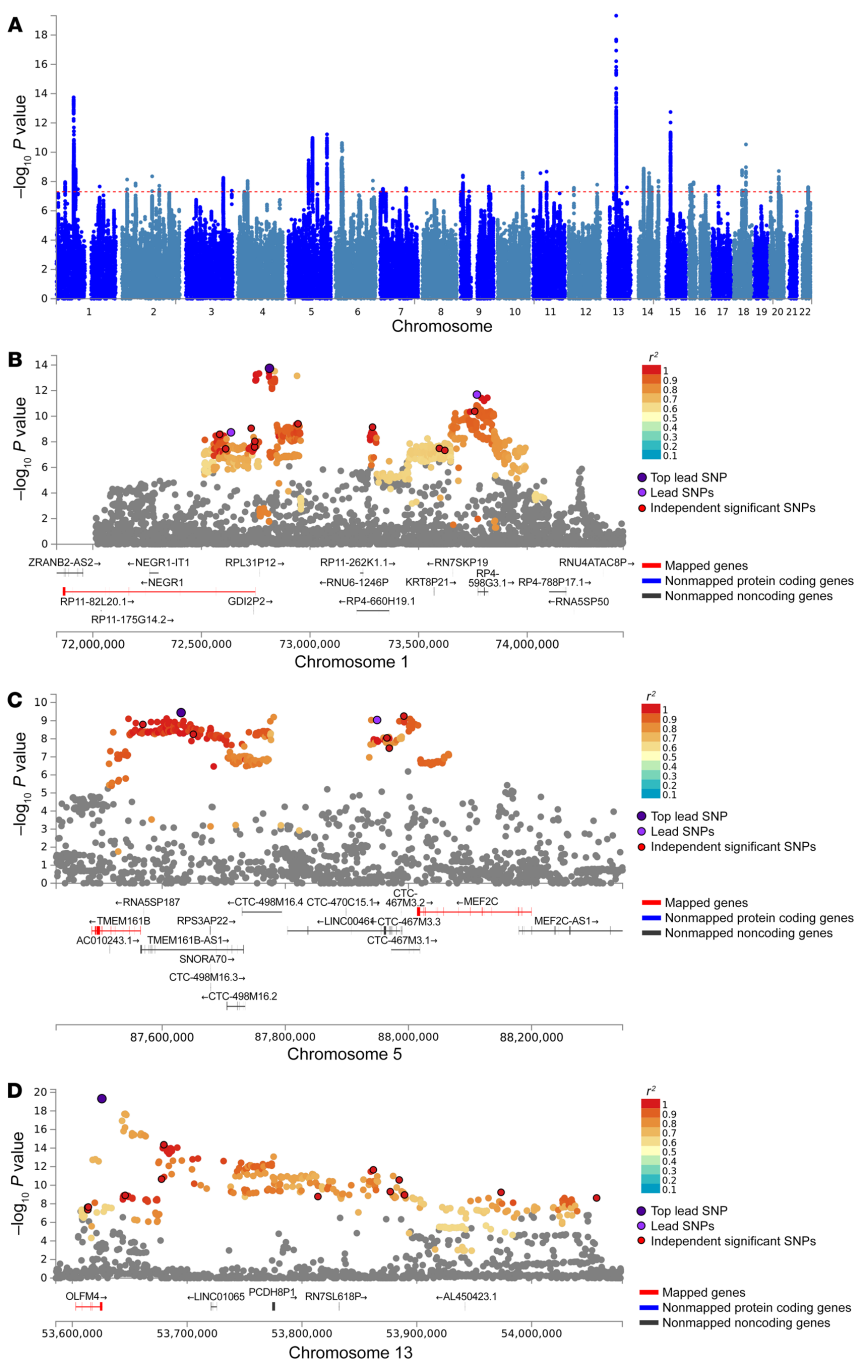
A meta-analysis of major depressive disorder (MDD) and posttraumatic stress disorder (PTSD). (**A**) Manhattan plot of meta-analysis of MDD with PTSD. The *x* axis is the chromosomal position of SNPs and the *y* axis is the significance of the SNPs (–log_10_[*P*]). (**B**–**D**) Three genomic loci. Each SNP is color coded based on its correlation (*r*^2^) with one of the independent significant SNPs (IndSigSNPs) if that correlation is greater than or equal to the *r*^2^ threshold of 0.6. Other SNPs (below an *r*^2^ of 0.6) are colored in gray. The top lead SNPs in genomic risk loci, lead SNPs, and IndSigSNPs are circled in black and colored in dark purple, purple, and red, respectively. Red lines: Genes mapped by positional mapping (mapped genes). Blue lines: Nonmapped protein-coding genes. Dark gray lines: Nonmapped, noncoding genes.

The cross-trait meta-analysis of MDD and PTSD revealed 47 loci harboring 111 independent significant SNPs (IndSigSNPs), and 53 lead SNPs, including 67 pleiotropic IndSigSNPs located in 29 loci associated with both traits (Figure 1D, Figure 3A, Table 2, and Supplemental Tables 2 and 3). IndSigSNPs were identified when their *P* values were genome-wide significant (*P* ≤ 5.0 × 10^−8^) and independent of each other (*r*^2^ < 0.6). Lead SNPs were identified as a subset of the IndSigSNPs that were in linkage disequilibrium (LD) with each other at *r*^2^ less than 0.1 within a 500-kb window. The association signals mapped to 1p31.1, 5q14.3, and 13q14.3 loci are shown in Figure 3, B–D. λMeta values were 1.09 ± 0.01, 1.16 ± 0.01, and 1.07 ± 0.01 between PTSD and MDD, DEP, and DAF, respectively, indicating no significant overlap of GWAS samples. Quantile-quantile plots of the observed meta-analysis statistics versus the expected statistics under the null model of no associations in the –log_10_(*P*) scale are shown in Supplemental Figure 1. According to tissue expression analysis, these associations were significantly enriched in brain tissues (Supplemental Figures 2 and 3).

A total of 91 protein-coding genes were identified at the *P*-value threshold of 5 × 10^–8^ (Supplemental Table 4). Of these genes, 67 (73.6%) were shared by the 2 disorders, and the remaining 24 were associated with MDD only. The genes identified in the present meta-analysis but not detected by the input GWAS were called “study-level” genes. The genes identified in the present meta-analysis but not reported by any previous GWAS for a given trait were called “trait-level” genes.

**Figure 4:**
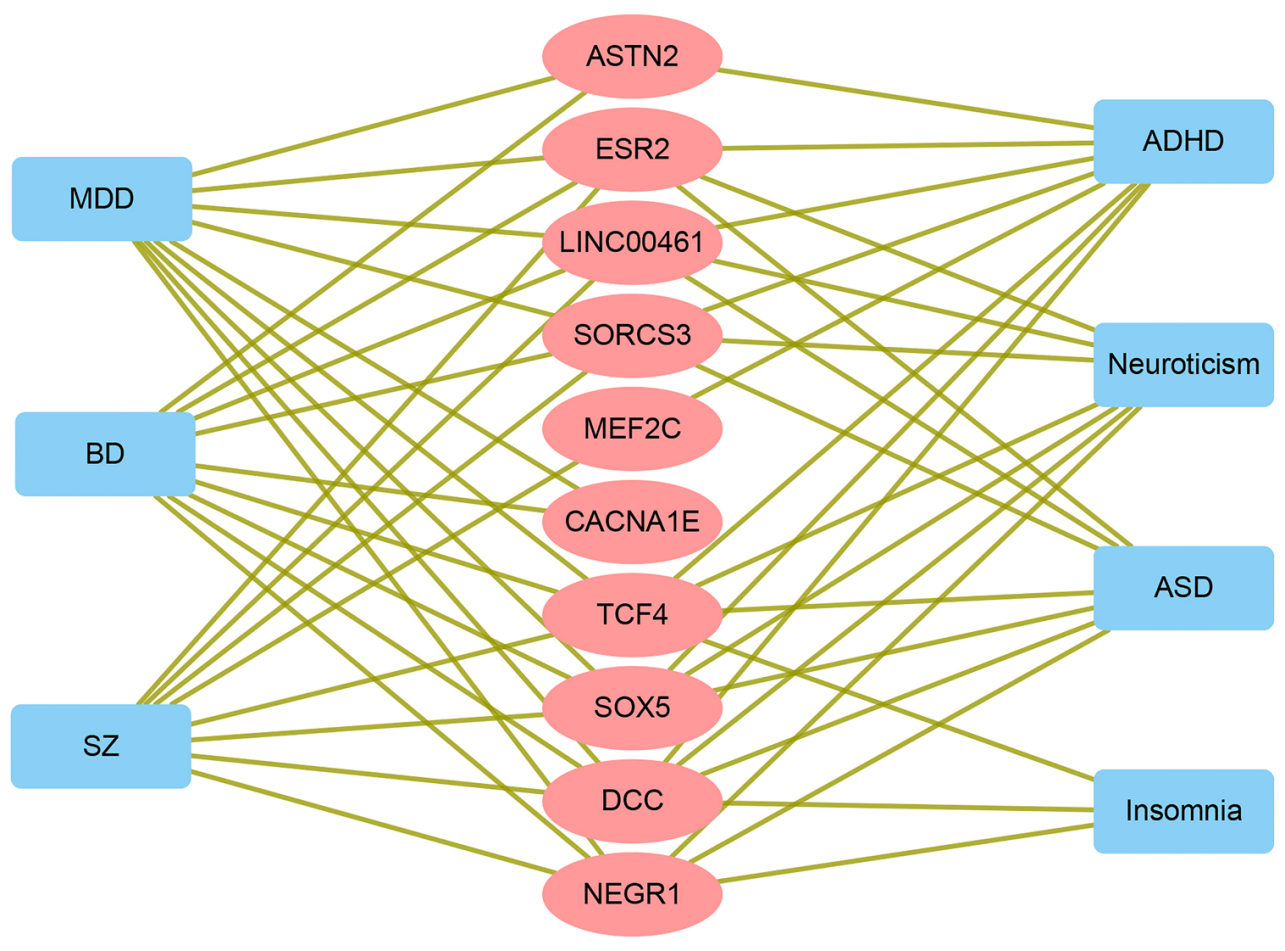
Shared genes between PTSD and MDD and their associations with common mental traits reported by previous GWASs. SZ, schizophrenia; MDD, major depressive disorder; BD, bipolar disorder; ASD, autism spectrum disorder; ADHD, attention deficit/hyperactivity disorder; PTSD, posttraumatic stress disorder.

Among the 91 genes associated with MDD, 74 genes were labeled as study-level, and 51 genes as trait-level. Twenty-four out of 74 study-level genes were reported for MDD by one or more previous GWASs. Among the 51 trait-level genes, 12 have been reported for their association with one or more of other mental traits, including *MEF2C*, *NKAPL*, and *EP300* (Supplemental Table 4). All 67 genes associated with PTSD were trait-level (Supplemental Table 4), of which 33 genes have been previously reported for one or more of the 7 mental traits (Supplemental Table 4). Some of the pleiotropic genes and their associated traits are described in Figure 4 and Supplemental Table 5.

## Discussion

Studies of PTSD have shown that the presence of comorbidity is the rule rather than the exception, with depressive disorder being the most commonly ascertained comorbid diagnosis. The construct of PTSD is widely debated because of its inherent controversy (13, 14). In particular, symptom overlap of PTSD with other psychiatric diagnoses, along with commonly detected comorbidity, raises concerns about its distinctive mental disorder nature.

Several explanations of comorbidity between PTSD and MDD have been suggested (15). One of the models posits that PTSD and MDD share common risk factors or vulnerabilities. Another model suggests that the detection of comorbidity is an artifact of symptom overlap (16). The relationship between PTSD and MDD has been explored previously with advanced statistical approaches. Confirmatory factor analysis and bifactor modeling may aid in partitioning a disease-specific variance from its shared variance. In this analysis, each item can load to a subscale factor and a general bifactor, or a general distress factor. A recent study employed confirmatory factor analyses and a bifactor model to show that the comorbidity between PTSD and MDD may be accounted for by the general distress factor (11), which represents a transdiagnostic component spanning many mental disorders (17, 18). Here we revealed a causal association between PTSD and MDD, extending the genetic foundation for the shared vulnerability between the 2 traits. The case of one disease encompassing another (or one disease being a subtype of another) may represent an extreme case of the shared vulnerability model.

As of now, the strongest genetic correlations between 2 psychiatric conditions were detected for bipolar disorder and schizophrenia, with the correlation coefficients being approximately 0.70 (19–21). At this time, however, it is already clear that all mental disorders are genetically intercorrelated and interconnected, thus suggesting that current diagnostic boundaries may not adequately reflect underlying etiology and emphasizing the need for further refinements of psychiatric nosology.

Given that the evidence supporting conceptual differentiation of MDD and PTSD is limited, here we attempted to examine underlying dimensions of these 2 psychopathological conditions. We have detected an extraordinarily high genetic correlation between MDD and PTSD (*r* = 0.80), which was higher than that for the bipolar disorder–schizophrenia correlations reported earlier. A similarly strong genetic correlation was also reported for PTSD and the depressive symptoms (*r* = 0.80) (22). These observations provide direct evidence supporting the closeness of MDD and PTSD, at a level inconceivable for 2 distinct nosological entities.

Polygenic overlap analysis indicates that MDD and DEP each possess twice-larger polygenic components than those of PTSD. Most intriguingly, the set of causal variants contributing to PTSD is all covered by the causal variants of MDD and DEP in its entirety, highlighting that the genetically determined component of PTSD is an integral part of MDD genetics. The complete overlap observed in this study is a strong argument against classifying MDD and PTSD cases as belonging to 2 distinctive disease categories. Instead, it suggests that PTSD may be a part of MDD, owing to their shared etiology.

MDD-PTSD overlap was detected in the course of the cross-trait meta-analysis at the genome-wide level, which had revealed a substantial overlap of the genomic loci contributing to MDD and PTSD, with 29 out of 30 genomic loci contributing to PTSD being shared with MDD. In other words, nearly all of the top risk signals for PTSD confer the risk for MDD as well. When the evidence is considered altogether, as an overlap of the causal variants, genomic loci, and risk genes, MDD and PTSD become inseparable, with a provision that MDD is influenced by a broader spectrum of causal gene variants than PTSD.

For both MDD and PTSD, the present analysis highlights a set of potentially novel risk genes, including some protein-coding ones. Among the 74 study-level genes, nearly one-third (24 out 74) replicated the signals observed in previous GWASs, which is unlikely to happen by chance (Fisher’s *P* = 4.89 × 10^–19^), given that the total number of protein-coding genes is 30,000. All 67 genes associated with PTSD were trait-level genes, of which 33 have been implicated in one or more other mental traits. This set of 67 PTSD-associated genes was significantly enriched in risk genes contributing to any of the 7 mental traits (Fisher’s *P* = 1.38 × 10^–19^). The results of the 2 enrichment tests support the validity of the meta-analysis findings presented here.

Additionally, we have identified a set of genes that are previously undescribed for PTSD. Detailed analysis of these genes may provide additional insights into the shared pathogenesis of the 2 illnesses, with some of the shared genes possibly contributing to the treatment response. Previous studies have reported only a limited number of genome-wide genes with a significant association with PTSD, including 35 protein-coding genes (GWAS Catalog). The results presented here greatly expand the current repertoire of the risk genes contributing to PTSD by adding the 67 identified genes to the set. The list of pleiotropic risk factors acting across a variety of psychiatric disorders includes such well-described candidates as *NEGR1*, *SOX5*, *SORCS3*, *DCC*, and *TCF4* and indicates that MDD and PTSD are part of the greater spectrum of mental disorders with shared genetic liability.

For further dissection, we concentrated on 3 particular loci that influence both MDD and PTSD. The 1p31.1 locus contains the pleiotropic gene *NEGR1*, well known for its contribution to a variety of mental disorders. It encodes neuronal growth regulator 1 (NEGR1), a member of the IgLON superfamily of cell adhesion molecules (23). NEGR1 is highly expressed in the cerebral cortex and hippocampus, suggesting its function in neurodevelopment (24, 25). In mice, a deficiency of *Negr1* shifts the ratio of excitatory/inhibitory neurons and influences adaptive behavioral profiles (26), thus indicating that its GWAS-confirmed involvement in a wide spectrum of psychiatric disorders has roots in the intrinsic function rather than in the colocalization with regulatory lncRNAs. Peculiarly, *NEGR1* variations were reported to be associated with both obesity and the response to treatment with selective serotonin reuptake inhibitors (SSRIs) (27). Moreover, in the cerebral cortex of rats, expression levels of *NEGR1* are affected by treatment with the common antidepressant venlafaxine (28).

Located within the 5q14.3 region, the *LINC00461*–*MEF2C* gene cluster is one of the most pleiotropic genomic regions contributing to many major psychiatric traits (29). The MEF2C protein plays a crucial role in the neuronal development of the neocortex, where its expression is abundant (30). In particular, it promotes the formation of neuronal synapses (31, 32), rescues neuronal cells from apoptosis (33), and regulates the differentiation and maturation of neural progenitors (34). *MEF2C* has been implicated in multiple neuropsychiatric phenotypes and disorders, including autism spectrum disorder, schizophrenia, and Alzheimer’s disease (35–39). Adjacent lncRNA *LINC00461* is also brain predominant, with its sequence and expression pattern being highly conserved across a diverse set of species (40).

The strongest association signal for PTSD was found on chromosome 13 (*P* = 4.79 × 10^–20^), in a region spanning noncoding mRNAs *LINC01065*, *PCDH8P1*, and *RN7SL618P* as well as olfactomedin 4–encoding gene *OLFM4*, which takes part in innate immunity, inflammation, and cancer. Even though this region has been repeatedly implicated in MDD and related phenotypes (41, 42), it remains understudied. A possibility of *OLFM4* involvement in cross-talk between the tissues of the gut-brain axis warrants future investigations.

It is important to note that observational epidemiological studies are subject to various biases resulting from confounding factors and reverse causation. The analytic framework of MR utilizes genetic variants as instrumental variables, thus allowing one to test for causative association between an exposure and an outcome. Here we employed MR analysis to evaluate the causal effects between PTSD and the depressive phenotypes. Our results indicate causal effects of the liability to the depressive phenotypes on PTSD, suggesting that the individuals carrying risk variants for the depressive phenotypes also have an increased risk for the development of PTSD. This finding is in line with the common observation that a history of MDD or DAF serves as a risk factor for the development of trauma-induced PTSD (5). On the other hand, the genetic liability to PTSD also increases the risk for DEP or DAF, but on a smaller scale than the effects of the depressive phenotypes on PTSD. This is understandable, given that a set of causal variants contributing to PTSD represents only half of a set contributing to MDD or DEP.

Taken together, our observations suggest that the 2 previously distinct disorders, PTSD and MDD, are one and that the detected differences largely reflect circumstances rather than intrinsic pathology. PTSD is the most common psychopathological outcome of exposure to trauma, while posttraumatic MDD diagnosis is second in prevalence (43). Upon exposure to trauma and depending on genetic and environmental circumstances, individuals with high liability may experience an onset of PTSD, MDD, or both.

Our data support the placement of PTSD into a larger category of MDD as its subtype. The results of this study may have important implications for refining or restructuring current psychiatric nosology. Since no pharmacotherapies specifically address PTSD, the notion that PTSD may be a subtype of MDD is consistent with the common pharmacological practice of administering antidepressants (44). Because of that, conceptualizing PTSD into the MDD spectrum will not lead to an oversimplification or a bias in clinical practice. From an analytic standpoint, integration of PTSD with MDD may lead to an acquisition of additional insights, including a set of novel genetic contributors, similar to ones revealed in the present cross-trait meta-analysis.

Since genetic variations are inherited, and, therefore, do not change with circumstances of one’s life, they serve as reliable, objective variables that represent the pathophysiological roots of a trait rather than its symptoms. Strengths of this study include the use of large GWAS data sets covering both PTSD and the depressive phenotypes and the deliberate limiting of studied populations to individuals of European ancestry. Hence, possible heterogeneity is reduced. Lastly, in the present study, the genetic relationships between PTSD and depressive phenotypes were discerned systematically by engaging multiple analytic frameworks. In light of some limitations, this study should be interpreted with caution. In particular, its focus on the genetic component of each trait leads us to the necessary exclusion of environmental components. Therefore, validation of our findings in additional data sets is warranted, especially in samples from other populations.

### Conclusions.

In summary, the multiple lines of evidence converge to support the notion that, from the point of view of a geneticist, PTSD may be a subtype of MDD. This inference may have implications for psychiatric nosology, and may lead to eventual improvement in the diagnosis and the treatment of psychiatric disorders.

## Methods

### GWAS summary data sets and quality control.

This study relied on summary-level data that have been made publicly available. Ethical approval had been obtained in all original studies. In addition, part of the MDD data set was obtained from 23andMe, after approval. The MDD data set, composed of 135,458 cases and 344,901 healthy controls, was derived from 7 case-control cohorts (45). A total of 44 loci were identified as associated with MDD (45). The DEP data set contained 246,363 cases and 561,190 controls from UK Biobank, 23andMe, and Psychiatric Genomics Consortium; its analysis led to identifying 102 loci (46). The DAF data set contained 357,957 participants from the UK Biobank (47). To obtain scores for the cluster-depressed affect, the sum of scores on 4 Eysenck Personality Questionnaire Revised Short Form items was utilized. The PTSD data set included 23,212 cases and 151,447 controls (22). PTSD was confirmed based either on lifetime (where possible) or current PTSD. All the patients were from the European population. For each data set, detailed descriptions and quality control are provided in the supplemental material.

### Genetic correlation and polygenic overlap analysis.

GWAS summary results were utilized to analyze the genetic correlation of MDD with PTSD using LD score regression software (LDSC, v1.0.1) (48, 49). Polygenic overlap was analyzed by MiXeR v1.3 using default parameters (12). In the MiXeR pipeline, total amounts of shared and trait-level causal variants across a pair of traits were estimated. The test statistics of MiXeR account for the effects of the LD structure, the minor allele frequency, the sample size, the cryptic relationships, and the sample overlap. The total amount of causal variants was reported as 22.6% of the total estimate, which covers 90% of SNP heritability for each trait.

### MR analysis.

Bidirectional causal associations between MDD and PTSD were inferred using GSMR v1.0.9 (50). Instrumental variants were selected based on default *P* less than or equal to 5 × 10^–8^. In an MR analysis, pleiotropy is a known source of inflated estimations (51), which necessitates the use of additional statistics. In GSMR, genetic instruments with apparent disease-specific or risk factor–specific pleiotropic effects are detected and eliminated by the HEIDI-outlier procedure (52). The intercept from the MR-Egger model was used as a measure of the directional pleiotropy (53).

### Cross-trait meta-analysis.

Using the subset-based fixed-effects method ASSET v2.4.0, which permits the characterization of each SNP with respect to the pattern of its effects on multiple phenotypes (54), we performed a cross-trait meta-analysis of MDD and PTSD. For each variant, a *P* value showing the best subset containing the studies contributing to the overall association signal was recorded. Each meta-analysis pooled the effects of a given SNP across *K* studies, weighting these effects by the size of the study. After subset-based meta-analysis, SNPs with *P* values less than 5 × 10^–8^ were considered statistically significant. FUMA was used for functional annotation and gene mapping of the variants and for identifying LD-independent genomic regions (55). For each outcome of the cross-trait meta-analysis, tissue enrichment was quantified by SNP-based analysis conducted in FUMA (55). To explore whether the genes highlighted by our meta-analysis have been previously identified in GWASs, we mined the GWAS Catalog database (https://www.ebi.ac.uk/gwas/) (56) for 7 common mental traits, including MDD, schizophrenia, bipolar disorder, autism spectrum disorder, attention deficit/hyperactivity disorder, neuroticism, and insomnia.

To ensure that sample overlap had not inflated the estimates of genetic overlap between PTSD and the depressive phenotypes, λmeta statistics were calculated (57). In calculating λmeta, sample overlap or heterogeneity is detected by measuring concordance of effect sizes. Under the null hypothesis, λmeta equals 1 when the pair of cohorts is completely independent. When samples overlap, λmeta is less than 1.

### Statistics.

This study estimated genetic correlation and polygenic overlap between PTSD and the depressive phenotypes by LD score regression and polygenic overlap analysis, investigated bidirectional causal relationships between MDD and the depressive phenotypes by 2-sample MR, and identified the pleiotropic genomic loci and the genes shared between MDD and PTSD by cross-trait meta-analysis (*P* < 5.0 × 10^–8^). All the statistical analyses were conducted in the R 3.6.1 or Python 3.7 environment. A detailed description of the methods is provided in the supplemental material. *P* values less than 0.05 were considered significant, and multiple testing was adjusted by false discovery rate (FDR).

### Study approval.

As the current study was based on published studies and public databases, no additional ethics approval or consent to participate was required.

## Author contributions

FZ conceived the study, analyzed the data, and wrote the manuscript. AB, SR, and MX contributed intellectually to data analysis and manuscript editing. XZ, QW, YX, JS, CW, JC, XX, NZ, LT, JY, GW, LC, and HC provided overall scientific support for the research project. The order of co–first authors was based on conceptual and intellectual contributions to the project.

## Supplementary Material

Supplemental data

Trial reporting checklists

ICMJE disclosure forms

## Figures and Tables

**Table 2:**
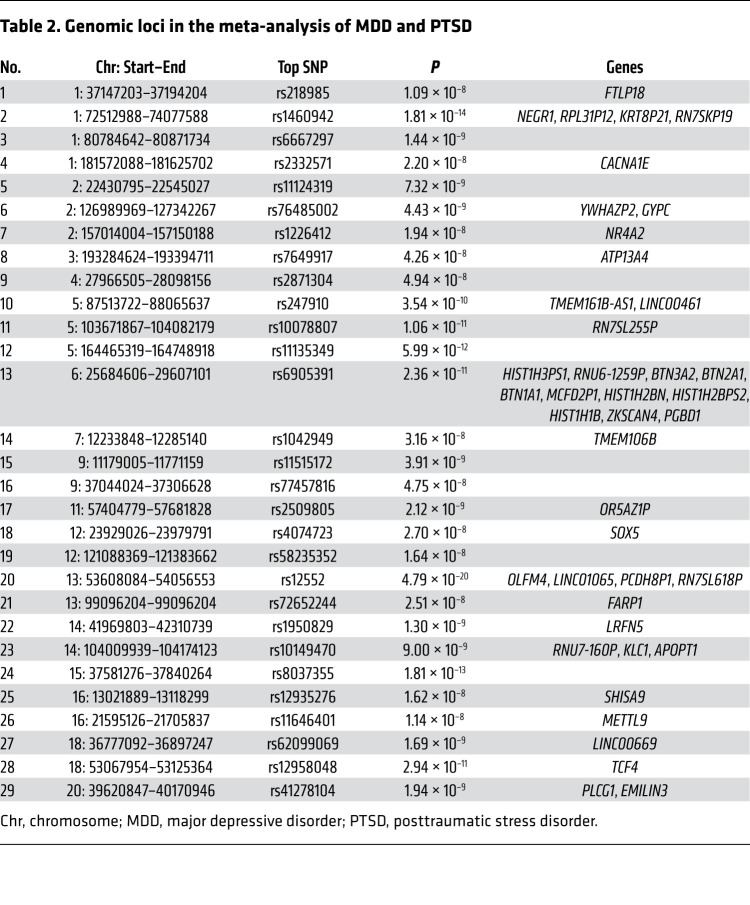
Genomic loci in the meta-analysis of MDD and PTSD

**Table 1:**
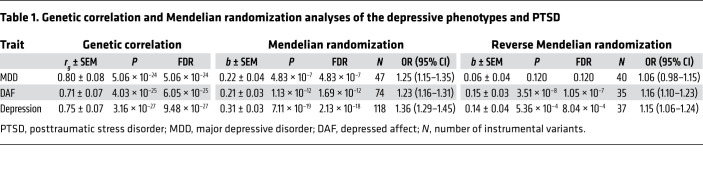
Genetic correlation and Mendelian randomization analyses of the depressive phenotypes and PTSD

## Version 1 04/27/2021

In-Press Preview

## Version 2 02/01/2022

Electronic publication
