# Supplementary material for: Genetic evidence suggests posttraumatic stress disorder as a subtype of major depressive disorder
Source: J Clin Invest. 2022 Feb 1;132(3):e145942. doi: 10.1172/JCI145942 (PMC8803333; doi:10.1172/JCI145942)
Supplement: Supplemental data [file jci-132-145942-s138.pdf]

Supplementary File

1. Methods

1.1 Dataset description and subject details

1.2 Quality control of GWAS datasets

1.3 LD score regression

1.4 Polygenic overlap analysis

1.5 Cross-trait meta-analysis

1.6 MR analysis

2. Supplementary Table 1. Pleiotropic effects of the causal effects between PTSD and the depressive phenotypes

3. Supplementary Table 2. Genomic loci of meta-analysis of MDD and PTSD

4. Supplementary Table 3. Independent significant SNPs of the meta-analysis of MDD and PTSD

5. Supplementary Table 4. Protein-coding genes identified by the meta-analysis shared by MDD and PTSD

6. Supplementary Table 5. Pleiotropic genes and their associations with the seven mental traits.

7. Supplementary Figure 1. Quantile-quantile plots of the observed meta-analysis statistics versus the expected statistics for MDD and PTSD.

8. Supplementary Figure 2. Gene property analysis for tissue specificity in GTEx tissues

9. Supplementary Figure 3. Gene property analysis for tissue specificity in general GTEx tissues

10. References

1. Methods

1.1 Dataset description and subject details

Major depressive disorder | Wray et al., 2018

The MDD dataset includes 135,458 cases and 344,901 controls from seven case-control cohorts. A total of 44 loci were identified as associated with major depression. The first cohort included 29 case-control samples of European descent where lifetime diagnosis of major depressive disorder was ascertained using structured clinical interviews (DSM-V, ICD-9, or ICD-10), clinician-administered checklists, or review of medical records. Six additional cohorts of European ancestry, including the Hyde et al. study (23andMe, Inc.), determined case status using other methods including national or hospital treatment registers, self-reported symptoms or treatment by a medical professional, or direct interviews.

Broad depression | Howard et al., 2019

The broad depression (DEP) dataset included 246,363 cases and 561,190 controls (1). A total of 102 loci were identified as associated with depression. All participants were of European origins, from UK Biobank, 23andMe, and Psychiatric Genomics Consortium (PGC).

UK Biobank. Within UK Biobank, the authors used the broad definition of depression (2). In summary, case and control status of broad depression was defined by the participants' response to the questions 'Have you ever seen a general practitioner for nerves, anxiety, tension or depression?' or 'Have you ever seen a psychiatrist for nerves, anxiety, tension or depression?'. Exclusions were applied to participants who were identified with bipolar disorder, schizophrenia, or personality disorder using self-declared data as well as prescriptions for antipsychotic medications. This provided a total of 127,552 cases and 233,763 controls (n=361,315, prevalence=0.353) for analysis.

23andMe. The authors obtained the genome-wide association study results from the discovery 23andMe subset (23andMe_307k) from the Hyde, et al.' analysis (3). Phenotypic status was based on responses to web-based surveys, with individuals that self-reported as having received a clinical diagnosis or treatment for depression classified as cases. This provided a total of 75,607 cases and 231,747 controls (n=307,354, prevalence=0.25). The authors excluded variants with an imputation accuracy threshold <0.6 and with a minor allele frequency <0.005, which left a total of 8,995,180 variants.

Major depressive disorder working group of the PGC. Wray et al. conducted a large meta-analysis of MDD (4), utilizing European-ancestry PGC cohorts with an emphasis placed on obtaining clinically-derived phenotypes for MDD. Their meta-analysis included the 23andMe_307k discovery cohort (3) and a previous release of the UK Biobank data (5). Howard et al. obtained the summary statistics from their meta-analysis of major depression with the 23andMe_307k and the previous UK Biobank cohorts removed (PGC_139k). This provided a total of 12,149,399 variant calls for 43,204 cases and 95,680 controls (n=138,884, prevalence=0.31). Howard et al. excluded variants with an imputation accuracy threshold<0.6 or a minor allele frequency<0.005, which left a total of 10,365,555 variants.

Depressed affect | Nagel et al., 2018

The sum of scores on four Eysenck Personality Questionnaire Revised Short Form (EPQ-RS) items (i.e., "Do you often feel lonely?", "Do you ever feel 'just miserable' for no reason?", "Does your mood often go up and down?", and "Do you often feel 'fed up'?") was used to obtain scores for the cluster depressed affect. In the item-cluster analyses, only participants with complete scores on all four items were included, which resulted in n=357,957 for depressed affect (6).

Posttraumatic stress disorder | Nievergelt et al. 2019

The PGC-PTSD Freeze 2 dataset (PGC2) includes 60 ancestrally diverse studies from Europe, Africa and the Americas. Summary result of European origin was used in the present study, 23,212 cases and 151,447 controls. PTSD assessment was based either on lifetime (where possible) or current PTSD (i.e. including participants with a potential lifetime PTSD diagnosis as controls), and PTSD diagnosis was established using various instruments and different versions of the DSM (DSM-III-R, DSM-IV, DSM-5). For GWAS analyses, all studies provided PTSD case status as determined using standard criteria and control subjects not meeting the PTSD diagnostic criteria. The majority of controls was trauma-exposed. A detailed description of the studies included is presented in Supplementary Methods of Nievergelt et al. (7). All subjects provided written informed consent and studies were approved by the relevant institutional review boards.

SNPs were compared between the two traits. If a SNP is mapped in opposite strands in the two datasets, alleles of the SNP in the second dataset were flipped. Effect direction of a SNP was reversed for the second dataset if alleles of the SNP on the contrary in the two datasets.

1.2 Quality control of GWAS datasets

We compared SNP alleles between MDD dataset and each of the CVD datasets. 1) SNPs were fileted based on INFO $\geqslant$0.80 if it exists. 2) Each SNP was compared between the two datasets and SNPs with conflicting alleles between each pair of datasets were excluded. 3) If an SNP was mapped to opposite strands in the two datasets, alleles of this SNP in the second dataset were flipped, and effect direction was reversed. For palindromic SNPs (with A/T or C/G alleles), the information on allele frequency was used to resolve strand ambiguity.

1.3 LD score regression

LD score regression software v1.0.1 were used to analyze the genetic correlation of MDD with PTSD. The 1000 Genome project phase 3(8) were used to estimate the LD structure, which was obtained from the LD score regression website(9-11). SNPs were filtered by 1.1 million variants, subset of 1000 Genomes and HapMap3(12), with MAF above 0.05, MHC and other long-range LD regions excluded.

1.4 Polygenic overlap analysis

Frei et al. introduced a novel statistical framework (MiXeR) to quantify polygenic overlap irrespective of genetic correlation between traits (13). In the MiXeR analysis, an effective sample size of Neff = 4/(1/Ncase + 1/Ncontrol) was used to account for imbalanced numbers of cases and controls. The python_convert (v0.9.2) pipeline was used to harmonize GWAS summary statistics (https://github.com/precimed/python_convert). Calculation of the LD structure, LD scores l_i and shape parameter η_j are based on 9,997,231 SNPs from 1000 Genomes Phase 3 data, downloaded from LD score regression website (9-11). The HEIDI-outlier (14) method is used to detect pleiotropic SNPs at which the estimates of b_{xy} are significantly different from expected under a causal model, and remove them from the GSMR analysis.

1.5 Cross-trait meta-analysis

ASSET meta-analysis is an agnostic approach that generalizes standard fixed-effects meta-analysis by allowing a subset of the input GWASs to have no effect on a given SNP. The method exhaustively explore all possible subsets of ‘‘non-null’’ GWAS inputs within a fixed-effect framework to identify the strongest association signal in both positive and negative directions. The cross-trait meta-analysis can be applied to binary traits or quantitative traits, but can’t be used in a mixture of binary traits and quantitative traits. Since DAF was a quantitative trait, we could not use it for the meta-analysis with MDD. Considering the MDD dataset used a more strict phenotype than DEP that used “broad depression” as its phenotype, we used MDD

rather than DEP for the meta-analysis. Only SNPs that were present for both the traits were retained as inputs to the ASSET meta-analysis. We carried out one-sided analysis by exploring models in which all non-null studies have effects in the same direction. Default parameters were applied with the ‘‘h.traits’’ function in ASSET.

FUMA was used to map SNPs to genes and identify LD-independent genomic regions.(15) Firstly, independent significant SNPs (IndSigSNPs) were identified on the basis of their *P*-value being genome-wide significant ($P \leq 5.0 \times 10^{-8}$) and being independent from each other ($r^2 < 0.6$). Secondly, Lead SNPs were identified as a subset of the independent significant SNPs that were in LD with each other at $r^2 < 0.1$ within a 500 kb window. Genomic risk loci were identified by merging lead SNPs if they were closer than 500 kb apart. Clumping procedures were carried out on the basis of the European 1000 Genomes Project phase 3 reference panel. Due to extensive LD, the MHC region was merged into one region (chr6:25-35Mb). Genes within 100 kb of each variant were mapped.

To identify tissue specificity of the phenotype, FUMA performs MAGMA (16) gene-property analyses to test relationships between tissue specific gene expression profiles and disease-gene associations. The gene-property analysis is based on the regression model,

$$Z \sim \beta_0 + E_t\beta_E + A\beta_A + B\beta_B + \epsilon$$

where Z is a gene-based Z-score converted from the gene-based P-value, B is a matrix of several technical confounders included by default. E t is the gene expression value of a testing tissue type c and A is the average expression across tissue types in a data set, defined as follows:

$$E_t = \sum_{i}^{n} log_2(e_i + 1)/n$$

$$A = \sum_{j \in T}^{N} E_j/N$$

where n is the number of samples in tissue type t, e_i is the expression value of a sample in the tissue type t (e.g. RPKM count or TPM), N is the number of tissue types in a data set and T = {tissue type 1, tissue type 2, ..., tissue type N}. It performed a one-sided test ($\beta_E > 0$) which is essentially testing the positive relationship between tissue specificity and genetic association of genes.

1.6 MR analysis

To infer credible causal associations between MDD and PTSD, we performed Mendelian randomization analysis using GSMR V1.0.9.(17) This method utilizes summary-level data to test for putative causal associations between a risk factor (exposure) and an outcome by using independent genomewide significant SNPs as instrumental variables as an index of the exposure. HEIDI outlier detection was used to filter genetic instruments that showed clear pleiotropic effects on the exposure phenotype and the outcome phenotype. We used a threshold P value of 0.01 for the outlier detection analysis in HEIDI, which removes 1% of SNPs by chance if there is no pleiotropic effect. We tested for bidirectional causation by repeating the analyses while switching the role of each correlated phenotype as an exposure and intelligence as the outcome. For each trait, we selected independent ($r^2 = 0.1$), genome-wide significant lead SNPs as instrumental variables in the analyses.

The method estimates a putative causal effect of the exposure on the outcome (b_{xy}) as a function of the relationship between the SNP's effects on the exposure (b_{zx}) and the SNP's effects on the outcome (b_{zy}), given the assumption that the effect of non-pleiotropic SNPs on an exposure (x) should be related to their effect on the outcome (y) in an independent sample only via mediation through the phenotypic causal

pathway (b_{xy}). The estimated causal effect coefficients (b_{xy}) are approximately equal to the natural log odds ratio for a case-control trait. An odds ratio of 2 can be interpreted as a doubled risk compared with the population prevalence of a binary trait for every standard deviation increase in the exposure trait.

2. Supplementary Table 1. Pleiotropic effects of the causal effects between PTSD and the depressive phenotypes

Exposure	Outcome	Egger_intercept	s.e.	P
DAF	PTSD	8.69E-04	2.66E-03	0.745
DEP	PTSD	5.86E-04	2.21E-03	0.792
MDD	PTSD	8.69E-04	3.81E-03	0.821
PTSD	DAF	1.46E-03	2.05E-03	0.482
PTSD	DEP	5.62E-03	3.49E-03	0.116
PTSD	MDD	2.42E-03	2.55E-03	0.348

DAF: depressed affect; DEP: depression; MDD: major depressive disorder; PTSD: posttraumatic stress disorder.

3. Supplementary Table 2. Genomic loci of meta-analysis of MDD and PTSD

No	Chr:Start-End	Top SNP	P	Genes	Trait
1	1:37147203-37194204	rs218985	1.09E-08	FTLP18	MDD,PTSD
2	1:72512988-74077588	rs1460942	1.81E-14	NEGR1,RPL31P12,KRT8P21,RN7SKP19	MDD,PTSD
3	1:80784642-80871734	rs6667297	1.44E-09		MDD,PTSD
4	1:90779266-90804261	rs4261101	3.37E-08	RNU6-695P	MDD
5	1:181572088-181625702	rs2332571	2.20E-08	CACNA1E	MDD,PTSD
6	2:22430795-22545027	rs11124319	7.32E-09		MDD,PTSD
7	2:57943567-58065936	rs11682175	1.35E-08		MDD
8	2:126989969-127342267	rs76485002	4.43E-09	YWHAZP2,GYPC	MDD,PTSD
9	2:157014004-157150188	rs1226412	1.94E-08	NR4A2	MDD,PTSD
10	3:157830916-158284681	rs7430565	5.60E-09	RSRC1	MDD
11	3:193284624-193394711	rs7649917	4.26E-08	ATP13A4	MDD,PTSD
12	4:27966505-28098156	rs2871304	4.94E-08		MDD,PTSD
13	4:41882601-42135019	rs34215985	9.18E-09	SLC30A9	MDD
14	5:87513722-88065637	rs247910	3.54E-10	TMEM161B-AS1,LINC00461	MDD,PTSD
15	5:103671867-104082179	rs10078807	1.06E-11	RN7SL255P	MDD,PTSD
16	5:124203948-124287196	rs116755193	1.41E-08		MDD
17	5:164465319-164748918	rs11135349	5.99E-12		MDD,PTSD
18	5:166985224-167055936	rs4869056	2.68E-08	TENM2	MDD
19	6:25684606-29607101	rs6905391	2.36E-11	HIST1H3PS1,RNU6-1259P,BTN3A2,BTN2A1,BTN1A1,MCFD2P1,HIST1H2BN,HIST1H2BPS2,HIST1H1B,ZKSCAN4,PGBD1	MDD,PTSD
20	6:157780424-157801753	rs34517852	8.76E-09		PTSD
21	7:12233848-12285140	rs1042949	3.16E-08	TMEM106B	MDD,PTSD
22	7:109097792-109197067	rs4730387	2.85E-08		MDD
23	9:11179005-11771159	rs11515172	3.91E-09		MDD,PTSD
24	9:37044024-37306628	rs77457816	4.75E-08		MDD,PTSD
25	9:119699096-119766782	rs7856424	2.18E-08	ASTN2	MDD
26	10:106529451-106768514	rs61867293	2.48E-09	SORCS3	MDD
27	11:31810298-31858991	rs1806153	2.69E-09	RCN1	MDD
28	11:57404779-57681828	rs2509805	2.12E-09	OR5AZ1P	MDD,PTSD
29	12:23929026-23979791	rs4074723	2.70E-08	SOX5	MDD,PTSD
30	12:121088369-121383662	rs58235352	1.64E-08		MDD,PTSD
31	13:53608084-54056553	rs12552	4.79E-20	OLFM4,LINC01065,PCDH8P1,RN7SL618P	MDD,PTSD
32	13:99096204-99096204	rs72652244	2.51E-08	FARP1	MDD,PTSD
33	14:41969803-42310739	rs1950829	1.30E-09	LRFN5	MDD,PTSD
34	14:64649894-64876545	rs915057	2.64E-09	SYNE2,ESR2,TEX21P,MTHFD1	MDD
35	14:75108290-75397764	rs2003490	2.45E-08	AREL1,RPS6KL1	MDD
36	14:104009939-104174123	rs10149470	9.00E-09	RNU7-160P,KLC1,APOPT1	MDD,PTSD

37	15:37581276-37840264	rs8037355	1.81E-13		MDD,PTSD
38	16:6303301-6343315	rs8063603	1.76E-08		MDD
39	16:7657673-7675142	rs11077206	3.09E-08	RBFOX1	MDD
40	16:13021889-13118299	rs12935276	1.62E-08	SHISA9	MDD,PTSD
41	16:21595126-21705837	rs11646401	1.14E-08	METTL9	MDD,PTSD
42	17:27360504-27576962	rs17727765	2.20E-08	PIPOX,CRYBA1	MDD
43	18:36777092-36897247	rs62099069	1.69E-09	LINC00669	MDD,PTSD
44	18:50555931-50807090	rs11663393	3.94E-08	DCC	MDD
45	18:53067954-53125364	rs12958048	2.94E-11	TCF4	MDD,PTSD
46	20:39620847-40170946	rs41278104	1.94E-09	PLCG1,EMILIN3	MDD,PTSD
47	22:41408754-41713111	rs5758268	2.45E-08	L3MBTL2	MDD

Chr: chromosome; BP: base position.

4. Supplementary Table 3. Independent significant SNPs of the meta-analysis of MDD and PTSD

No	Locus	rsID	Chr:BP	P	OR (95%CI)	Trait	Genes
1	1	rs218985	1:37169665	1.09E-08	1.01 (1.01-1.02)	MDD,PTSD	FTLP18, RP4-614N24.1
2	2	rs2630400	1:72583763	2.59E-09	0.98 (0.97-0.99)	MDD	NEGR1
3	2	rs1545933	1:72611715	3.54E-08	1.03 (1.02-1.04)	MDD	NEGR1
4	2	rs12134600	1:72635996	1.82E-09	1.02 (1.01-1.03)	MDD,PTSD	NEGR1
5	2	rs1620977	1:72729142	8.80E-10	1.02 (1.01-1.02)	MDD,PTSD	NEGR1
6	2	rs1870676	1:72744996	2.53E-08	0.98 (0.97-0.99)	MDD	NEGR1
7	2	rs34579341	1:72745962	9.13E-09	1.03 (1.02-1.04)	MDD	NEGR1
8	2	rs1460942	1:72813256	1.81E-14	0.97 (0.97-0.98)	MDD	RPL31P12
9	2	rs7550173	1:72944617	3.91E-10	0.98 (0.97-0.98)	MDD	
10	2	rs12136862	1:73287890	7.12E-10	0.99 (0.98-0.99)	MDD,PTSD	RP4-660H19.1
11	2	rs11210135	1:73595371	3.25E-08	1.02 (1.01-1.03)	MDD	KRT8P21, RN7SKP19
12	2	rs112982912	1:73620750	4.67E-08	1.03 (1.02-1.04)	MDD,PTSD	KRT8P21, RN7SKP19
13	2	rs7523829	1:73758117	4.17E-11	1.01 (1.01-1.02)	MDD,PTSD	RP4-598G3.1
14	2	rs12129573	1:73768366	2.05E-12	1.02 (1.01-1.02)	MDD,PTSD	RP4-598G3.1
15	3	rs6667297	1:80795989	1.44E-09	0.98 (0.98-0.99)	MDD,PTSD	AL606519.1
16	4	rs4261101	1:90796053	3.37E-08	0.98 (0.97-0.99)	MDD	RNU6-695P
17	5	rs2332571	1:181625702	2.20E-08	1.01 (1.01-1.02)	MDD,PTSD	CACNA1E
18	6	rs11124319	2:22435356	7.32E-09	0.99 (0.98-0.99)	MDD,PTSD	AC068490.2
19	7	rs11682175	2:57987593	1.35E-08	0.98 (0.97-0.99)	MDD	CTD-2026C7.1
20	8	rs34795950	2:127171991	4.53E-08	0.98 (0.98-0.99)	MDD,PTSD	AC023347.1
21	8	rs76485002	2:127342267	4.43E-09	1.06 (1.04-1.08)	MDD	YWHAZP2, GYPC
22	9	rs1226412	2:157111313	1.94E-08	1.01 (1.01-1.02)	MDD,PTSD	NR4A2
23	10	rs7430565	3:158107180	5.60E-09	0.98 (0.97-0.99)	MDD	RSRC1
24	11	rs7649917	3:193289549	4.26E-08	0.99 (0.98-0.99)	MDD,PTSD	ATP13A4, RP11-175P19.2
25	12	rs2871304	4:27983557	4.94E-08	0.99 (0.98-0.99)	MDD,PTSD	AC007106.1
26	13	rs34215985	4:42047778	9.18E-09	0.98 (0.97-0.98)	MDD	SLC30A9
27	14	rs768705	5:87568710	1.60E-09	0.98 (0.97-0.98)	MDD	TMEM161B-AS1
28	14	rs247910	5:87630769	3.54E-10	0.98 (0.97-0.98)	MDD	TMEM161B-AS1
29	14	rs11748762	5:87650585	5.62E-09	1.04 (1.02-1.05)	MDD	TMEM161B-AS1
30	14	rs35267052	5:87949118	9.08E-10	0.98 (0.97-0.98)	MDD,PTSD	LINC00461
31	14	rs6893807	5:87965021	8.94E-09	0.97 (0.96-0.98)	MDD	LINC00461
32	14	rs6882046	5:87968864	3.31E-08	0.98 (0.97-0.99)	MDD	LINC00461
33	14	rs27732	5:87992576	5.52E-10	1.02 (1.01-1.03)	MDD	CTC-467M3.1
34	15	rs349588	5:103671867	2.91E-09	0.99 (0.98-0.99)	MDD,PTSD	RN7SL255P, RP11-6N13.1
35	15	rs1442114	5:103717385	1.69E-08	0.99 (0.98-0.99)	MDD,PTSD	RP11-6N13.1
36	15	rs12658032	5:103904226	3.02E-11	1.02 (1.01-1.02)	MDD,PTSD	RP11-6N13.1
37	15	rs10078807	5:103912319	1.06E-11	1.01 (1.01-1.02)	MDD,PTSD	RP11-6N13.1
38	16	rs116755193	5:124251883	1.41E-08	0.98 (0.97-0.99)	MDD	RP11-284A20.2, RP11-284A20.1
39	17	rs6883641	5:164475171	3.20E-09	1.01 (1.01-1.02)	MDD,PTSD	CTC-340A15.2
40	17	rs11135349	5:164523472	5.99E-12	0.98 (0.98-0.99)	MDD,PTSD	CTC-340A15.2
41	17	rs4312906	5:164642141	2.95E-08	1.01 (1.01-1.02)	MDD,PTSD	CTC-340A15.2, CTB-181F24.1

42	18	rs4869056	5:166992078	2.68E-08	0.98 (0.97-0.99)	MDD	TENM2
43	19	rs4711107	6:26342749	1.39E-08	1.01 (1.01-1.02)	MDD,PTSD	HIST1H3PS1, RNU6-1259P
44	19	rs9379850	6:26343057	1.13E-09	1.02 (1.01-1.02)	MDD,PTSD	HIST1H3PS1, RNU6-1259P
45	19	rs2073529	6:26375159	9.69E-10	1.02 (1.01-1.03)	MDD,PTSD	BTN3A2
46	19	rs13195402	6:26463575	9.93E-10	0.98 (0.97-0.98)	MDD,PTSD	BTN2A1
47	19	rs2273558	6:26466035	3.50E-09	1.02 (1.01-1.03)	MDD	BTN2A1
48	19	rs6926677	6:26478825	3.71E-10	0.98 (0.98-0.99)	MDD,PTSD	BTN2A1, BTN1A1
49	19	rs2064219	6:27376001	5.35E-10	1.02 (1.02-1.03)	MDD	MCFD2P1
50	19	rs143099908	6:27667247	4.84E-08	0.96 (0.95-0.98)	MDD,PTSD	RP1-15D7.1, RP1-97D16.1
51	19	rs200986	6:27824766	2.79E-08	0.97 (0.96-0.98)	MDD	HIST1H2BN, HIST1H2BPS2
52	19	rs200949	6:27835435	3.12E-10	1.02 (1.01-1.03)	MDD,PTSD	HIST1H1B
53	19	rs17720293	6:28214698	2.04E-08	0.98 (0.97-0.99)	MDD,PTSD	ZKSCAN4
54	19	rs1736904	6:28219270	3.28E-10	0.98 (0.98-0.99)	MDD,PTSD	ZKSCAN4
55	19	rs6905391	6:28262686	2.36E-11	0.98 (0.97-0.98)	MDD,PTSD	PGBD1
56	19	rs144447022	6:29244219	2.37E-10	0.98 (0.97-0.98)	MDD,PTSD	XXbac-BPG308J9.3
57	20	rs34517852	6:157789333	8.76E-09	1.04 (1.03-1.06)	PTSD	RP5-933K21.2, RP3-395C13.1
58	21	rs1042949	7:12272116	3.16E-08	0.99 (0.98-0.99)	MDD,PTSD	TMEM106B
59	22	rs4730387	7:109100414	2.85E-08	1.02 (1.01-1.03)	MDD	AC073071.1
60	23	rs11515172	9:11256041	3.91E-09	1.02 (1.01-1.02)	MDD,PTSD	RP11-23D5.1
61	23	rs10959913	9:11544964	1.26E-08	1.02 (1.01-1.03)	MDD	
62	24	rs77457816	9:37067726	4.75E-08	1.04 (1.02-1.05)	MDD,PTSD	RP11-297B17.2, RP11-465M18.1
63	25	rs7856424	9:119733595	2.18E-08	0.98 (0.97-0.99)	MDD	ASTN2
64	26	rs61867293	10:106563924	2.48E-09	0.97 (0.97-0.98)	MDD	SORCS3
65	26	rs1021363	10:106610839	3.01E-08	1.02 (1.01-1.03)	MDD	SORCS3
66	27	rs1806153	11:31850105	2.69E-09	1.02 (1.02-1.03)	MDD	RCN1
67	28	rs2509805	11:57650796	2.12E-09	1.02 (1.01-1.02)	MDD,PTSD	RP11-734C14.2, OR5AZ1P
68	29	rs4074723	12:23947737	2.70E-08	0.99 (0.98-0.99)	MDD,PTSD	SOX5
69	30	rs58235352	12:121186246	1.64E-08	0.97 (0.96-0.98)	MDD,PTSD	RP11-173P15.7
70	31	rs3803259	13:53613331	2.41E-08	0.99 (0.98-0.99)	MDD,PTSD	OLFM4
71	31	rs9568797	13:53613559	4.38E-08	0.99 (0.98-0.99)	MDD,PTSD	OLFM4
72	31	rs8181889	13:53613990	2.23E-08	0.99 (0.98-0.99)	MDD,PTSD	OLFM4
73	31	rs12552	13:53625781	4.79E-20	1.02 (1.02-1.02)	MDD,PTSD	OLFM4
74	31	rs2806948	13:53646075	1.24E-09	0.99 (0.98-0.99)	MDD,PTSD	OLFM4, LINC01065
75	31	rs2806985	13:53677723	2.23E-11	1.02 (1.02-1.03)	MDD	OLFM4, LINC01065
76	31	rs1951722	13:53679747	4.34E-15	1.02 (1.01-1.02)	MDD,PTSD	OLFM4, LINC01065
77	31	rs1407686	13:53813901	1.66E-09	1.01 (1.01-1.02)	MDD,PTSD	PCDH8P1, RN7SL618P
78	31	rs7326087	13:53862161	2.19E-12	0.98 (0.98-0.99)	MDD,PTSD	RN7SL618P, AL450423.1
79	31	rs4525388	13:53876897	4.92E-10	1.01 (1.01-1.02)	MDD,PTSD	RN7SL618P, AL450423.1
80	31	rs9527060	13:53884670	2.71E-11	1.02 (1.02-1.03)	MDD	RN7SL618P, AL450423.1
81	31	rs9563152	13:53889343	1.11E-09	1.02 (1.01-1.02)	MDD,PTSD	RN7SL618P, AL450423.1
82	31	rs1373273	13:53973351	5.83E-10	0.99 (0.98-0.99)	MDD,PTSD	AL450423.1
83	31	rs9596810	13:54056553	2.36E-09	0.99 (0.98-0.99)	MDD,PTSD	
84	32	rs72652244	13:99096204	2.51E-08	1.01 (1.01-1.02)	MDD,PTSD	FARP1

85	33	rs1950829	14:42097937	1.30E-09	1.01 (1.01-1.02)	MDD,PTSD	LRFN5
86	33	rs7143983	14:42215426	3.86E-08	0.98 (0.98-0.99)	MDD,PTSD	LRFN5
87	34	rs915057	14:64686207	2.64E-09	0.98 (0.97-0.99)	MDD	SYNE2, ESR2
88	34	rs1256112	14:64814311	2.18E-08	1.02 (1.01-1.03)	MDD	TEX21P
89	34	rs34110441	14:64876545	2.75E-08	0.98 (0.97-0.99)	MDD	MTHFD1
90	35	rs1045430	14:75130235	3.21E-08	0.98 (0.97-0.99)	MDD	AREL1
91	35	rs2003490	14:75377555	2.45E-08	1.02 (1.01-1.03)	MDD	RPS6KL1
92	36	rs10149470	14:104017953	9.00E-09	0.99 (0.98-0.99)	MDD,PTSD	RNU7-160P, RP11-894P9.2
93	36	rs55825782	14:104039132	2.30E-08	1.02 (1.01-1.02)	MDD,PTSD	KLC1, APOPT1, RP11-73M18.2
94	37	rs12102100	15:37587461	2.01E-10	1.01 (1.01-1.02)	MDD,PTSD	RP11-597G23.1, RP11-720L8.1
95	37	rs8037355	15:37643831	1.81E-13	0.98 (0.98-0.99)	MDD,PTSD	RP11-597G23.1, RP11-720L8.1
96	37	rs4923747	15:37754903	5.68E-12	1.02 (1.01-1.02)	MDD,PTSD	RP11-720L8.1
97	37	rs4924163	15:37772709	2.31E-08	1.01 (1.01-1.02)	MDD,PTSD	RP11-720L8.1
98	38	rs8063603	16:6310645	1.76E-08	0.98 (0.97-0.99)	MDD	RP11-420N3.2
99	39	rs11077204	16:7667187	4.26E-08	0.98 (0.97-0.99)	MDD	RBFOX1
100	39	rs11077206	16:7667332	3.09E-08	1.02 (1.01-1.03)	MDD	RBFOX1
101	40	rs12935276	16:13038723	1.62E-08	0.99 (0.98-0.99)	MDD,PTSD	SHISA9
102	41	rs11646401	16:21609978	1.14E-08	0.99 (0.98-0.99)	MDD,PTSD	METTL9
103	42	rs75581564	17:27363750	4.44E-08	1.03 (1.02-1.04)	MDD	PIPOX
104	42	rs17727765	17:27576962	2.20E-08	0.96 (0.95-0.98)	MDD	CRYBA1
105	43	rs4408608	18:36827566	4.06E-08	0.99 (0.98-0.99)	MDD,PTSD	LINC00669
106	43	rs62099069	18:36883737	1.69E-09	0.99 (0.98-0.99)	MDD,PTSD	LINC00669
107	44	rs11663393	18:50614732	3.94E-08	1.02 (1.01-1.03)	MDD	DCC
108	45	rs12958048	18:53101598	2.94E-11	1.01 (1.01-1.02)	MDD,PTSD	TCF4
109	46	rs6072299	20:39806772	2.28E-08	0.98 (0.98-0.99)	MDD,PTSD	PLCG1
110	46	rs41278104	20:39990700	1.94E-09	0.98 (0.98-0.99)	MDD,PTSD	EMILIN3
111	47	rs5758268	22:41622419	2.45E-08	0.98 (0.97-0.99)	MDD	L3MBTL2

5. Supplementary Table 4. Protein-coding genes identified by the meta-analysis of MDD and PTSD

**Symbol**	**Chr:start-end**	**minP**	**Locus**	**Traits**	**MDD_GWAS**	**PTSD_GWAS**	**MDD_GWASs**	**PTSD_GWASs**	**Multi_GWASs**
NEGR1	1:71861623-72748417	5.40E-14	2	MDD,PTSD	0	0	1	0	1
CACNA1E	1:181382238-181777219	2.20E-08	5	MDD,PTSD	0	0	1	0	1
RSRC1	3:157823644-158263519	5.60E-09	10	MDD	1	0	1	0	1
ATP13A4	3:193119866-193310900	4.26E-08	11	MDD,PTSD	0	0	1	0	1
SLC30A9	4:41992489-42092474	9.18E-09	13	MDD	1	0	1	0	1
BEND4	4:42112955-42154895	1.39E-08	13	MDD	0	0	1	0	1
TMEM161B	5:87485450-87565293	7.80E-10	14	MDD	0	0	1	0	1
MEF2C	5:88013975-88199922	1.06E-09	14	MDD	0	0	0	0	1
TENM2	5:166711804-167691162	2.68E-08	18	MDD	1	0	1	0	1
BTN3A2	6:26365387-26378546	9.69E-10	19	MDD,PTSD	0	0	1	0	1
BTN2A2	6:26383324-26395102	9.69E-10	19	MDD,PTSD	0	0	0	0	1
BTN3A1	6:26402465-26415444	2.24E-08	19	MDD,PTSD	0	0	1	0	1
BTN3A3	6:26440700-26453643	9.93E-10	19	MDD,PTSD	0	0	0	0	0
BTN2A1	6:26458150-26476849	3.71E-10	19	MDD,PTSD	0	0	1	0	1
BTN1A1	6:26501449-26510650	5.78E-10	19	MDD,PTSD	0	0	0	0	0
HMGN4	6:26538633-26546482	1.06E-09	19	MDD,PTSD	0	0	0	0	1
ZNF391	6:27342394-27371683	5.35E-10	19	MDD,PTSD	0	0	0	0	1
ZNF184	6:27418522-27440897	5.55E-10	19	MDD,PTSD	0	0	1	0	1
HIST1H2BL	6:27775257-27775709	2.40E-09	19	MDD,PTSD	0	0	0	0	0
HIST1H2AI	6:27775899-27776429	2.40E-09	19	MDD,PTSD	0	0	0	0	0
HIST1H3H	6:27777842-27778314	2.40E-09	19	MDD,PTSD	0	0	0	0	0
HIST1H2AJ	6:27782112-27782607	2.40E-09	19	MDD,PTSD	0	0	0	0	0
HIST1H2BM	6:27782822-27783267	2.36E-09	19	MDD,PTSD	0	0	0	0	0
HIST1H4J	6:27791884-27792257	1.70E-09	19	MDD,PTSD	0	0	0	0	0
HIST1H4K	6:27798952-27799305	1.70E-09	19	MDD,PTSD	0	0	0	0	0
HIST1H2AK	6:27805658-27806117	1.70E-09	19	MDD,PTSD	0	0	0	0	0
HIST1H2BN	6:27806323-27823487	1.37E-09	19	MDD,PTSD	0	0	0	0	0
HIST1H2AL	6:27833034-27833606	3.12E-10	19	MDD,PTSD	0	0	0	0	0
HIST1H1B	6:27834570-27835359	3.12E-10	19	MDD,PTSD	0	0	0	0	0
HIST1H3I	6:27839623-27840099	3.12E-10	19	MDD,PTSD	0	0	0	0	0
HIST1H4L	6:27840926-27841289	3.12E-10	19	MDD,PTSD	0	0	0	0	0
HIST1H3J	6:27858093-27860884	2.22E-09	19	MDD,PTSD	0	0	0	0	0
HIST1H2AM	6:27860477-27860963	2.22E-09	19	MDD,PTSD	0	0	0	0	0
HIST1H2BO	6:27861203-27861669	2.22E-09	19	MDD,PTSD	0	0	0	0	0

OR2B2	6:27878963-27880174	7.96E-09	19	MDD,PTSD	0	0	0	0	0
OR2B6	6:27925019-27925960	8.97E-09	19	MDD,PTSD	0	0	0	0	0
ZNF165	6:28048753-28057341	3.39E-09	19	MDD,PTSD	0	0	0	0	0
ZSCAN16	6:28092338-28097860	4.94E-09	19	MDD,PTSD	0	0	0	0	0
ZKSCAN8	6:28109688-28127250	5.07E-09	19	MDD,PTSD	0	0	0	0	0
ZSCAN9	6:28192664-28201260	6.61E-09	19	MDD,PTSD	0	0	0	0	1
ZKSCAN4	6:28212401-28227011	3.28E-10	19	MDD,PTSD	0	0	0	0	1
NKAPL	6:28227098-28228736	3.28E-10	19	MDD,PTSD	0	0	0	0	1
PGBD1	6:28249314-28270326	2.36E-11	19	MDD,PTSD	0	0	0	0	0
ZSCAN31	6:28292470-28324048	3.57E-11	19	MDD,PTSD	0	0	1	0	1
ZKSCAN3	6:28317691-28336947	4.19E-09	19	MDD,PTSD	0	0	1	0	1
ZSCAN12	6:28346732-28367511	3.02E-09	19	MDD,PTSD	0	0	1	0	1
ZSCAN23	6:28399707-28411279	2.97E-09	19	MDD,PTSD	0	0	0	0	1
TMEM106B	7:12250867-12282993	3.16E-08	21	MDD,PTSD	1	0	1	0	1
ASTN2	9:119187504-120177348	2.18E-08	25	MDD	1	0	1	0	1
SORCS3	10:106400859-107024993	2.48E-09	26	MDD	1	0	1	0	1
RCN1	11:31833939-32127301	2.69E-09	27	MDD	0	0	0	0	0
CLP1	11:57416465-57429340	1.61E-08	28	MDD,PTSD	0	0	0	0	0
ZDHHC5	11:57435219-57468659	1.61E-08	28	MDD,PTSD	0	0	1	0	1
MED19	11:57471186-57479693	2.64E-08	28	MDD,PTSD	0	0	0	0	0
TMX2	11:57480072-57508445	1.79E-08	28	MDD,PTSD	0	0	0	0	1
TMX2-CTNND1	11:57480077-57559058	1.79E-08	28	MDD,PTSD	0	0	0	0	0
C11orf31	11:57508825-57510986	2.90E-08	28	MDD,PTSD	0	0	0	0	0
BTBD18	11:57510986-57519253	2.44E-08	28	MDD,PTSD	0	0	0	0	0
CTNND1	11:57520715-57587018	1.85E-08	28	MDD,PTSD	0	0	1	0	1
SOX5	12:23682440-24103966	2.70E-08	29	MDD,PTSD	1	0	1	0	1
ACADS	12:121163538-121177811	1.64E-08	30	MDD,PTSD	0	0	0	0	0
OLFM4	13:53602894-53626192	4.79E-20	31	MDD,PTSD	1	0	1	0	1
FARP1	13:98794816-99102027	2.51E-08	32	MDD,PTSD	0	0	0	0	1
STK24	13:99102455-99230194	2.51E-08	32	MDD,PTSD	0	0	1	0	1
LRFN5	14:42076773-42373752	1.30E-09	33	MDD,PTSD	1	0	1	0	1
SYNE2	14:64319683-64693165	2.64E-09	34	MDD	1	0	1	0	1
ESR2	14:64550950-64804830	2.64E-09	34	MDD	1	0	1	0	1
MTHFD1	14:64854749-64926722	4.96E-09	34	MDD	0	0	0	0	0
AREL1	14:75120140-75179818	3.21E-08	35	MDD	0	0	1	0	1
DLST	14:75348594-75370448	2.45E-08	35	MDD	0	0	0	0	0
RPS6KL1	14:75370657-75390099	2.45E-08	35	MDD	0	0	1	0	1
BAG5	14:104022881-104029168	9.00E-09	36	MDD,PTSD	0	0	0	0	0
KLC1	14:104028233-104167888	2.30E-08	36	MDD,PTSD	0	0	1	0	1
APOPT1	14:104029299-10407386	2.30E-08	36	MDD,PTSD	0	0	0	0	0

	0								
RBFOX1	16:6069095-7763340	1.76E-08	38:39	MDD	1	0	1	0	1
SHISA9	16:12995477-13334272	1.62E-08	40	MDD,PTSD	1	0	1	0	1
METTL9	16:21608539-21668794	1.14E-08	41	MDD,PTSD	0	0	1	0	1
IGSF6	16:21652609-21663981	1.33E-08	41	MDD,PTSD	0	0	0	0	0
PIPOX	17:27277531-27384234	4.44E-08	42	MDD	0	0	1	0	1
MYO18A	17:27400528-27507430	2.80E-08	42	MDD	0	0	1	0	1
CRYBA1	17:27573881-27581512	2.20E-08	42	MDD	1	0	1	0	1
NUFIP2	17:27582854-27621136	2.20E-08	42	MDD	0	0	0	0	0
DCC	18:49866542-51057784	3.94E-08	44	MDD	1	0	1	0	1
TCF4	18:52889562-53332018	2.94E-11	45	MDD,PTSD	1	0	1	0	1
PLCG1	20:39765600-39811629	2.28E-08	46	MDD,PTSD	0	0	1	0	1
ZHX3	20:39807088-39946312	2.28E-08	46	MDD,PTSD	0	0	1	0	1
LPIN3	20:39969560-39989222	1.94E-09	46	MDD,PTSD	0	0	0	0	0
EMILIN3	20:39988606-39995467	1.94E-09	46	MDD,PTSD	0	0	1	0	1
EP300	22:41487790-41576081	4.27E-08	47	MDD	0	0	0	0	1
L3MBTL2	22:41601209-41627275	2.45E-08	47	MDD	1	0	1	0	1
CHADL	22:41625517-41636938	2.45E-08	47	MDD	0	0	0	0	0

MDD_GWAS: if the gene was implicated in the MDD GWAS; PTSD_GWAS: if the gene was implicated in the PTSD GWAS; MDD_GWASs: if the gene was implicated in all previous MDD GWASs (from GWAS catalog); PTSD_GWASs: if the gene was implicated in all previous PTSD GWASs (from GWAS catalog); Multi_GWASs: if the gene was implicated in the seven mental traits (MDD, schizophrenia, bipolar disorder, autism spectrum disorder, attention deficit/hyperactivity disorder, neuroticism, and insomnia).

6. Supplementary Table 5. Pleiotropic genes and their associations with the seven mental traits.

PUBMEDID	GENE	DISEASE/TRAIT
30804558	LINC00461	Autism spectrum disorder
30804558	NEGR1	Autism and major depressive disorder (MTAG)
30804558	SORCS3	Autism and major depressive disorder (MTAG)
28540026	TCF4	Autism spectrum disorder or schizophrenia
28540026	TCF4	Autism spectrum disorder or schizophrenia
23453885	RNA5SP459 - TCF4	Five mental traits
23453885	TCF4	Five mental traits
31835028	SOX5	Eight mental traits
31835028	LINC00461	Eight mental traits
31835028	DCC	Eight mental traits
31835028	TCF4	Eight mental traits
31835028	SORCS3	Eight mental traits
31835028	LINC00461	Eight mental traits
31835028	SORCS3	Eight mental traits
31835028	SOX5	Eight mental traits
31835028	SYNE2, ESR2	Eight mental traits
31835028	TCF4	Eight mental traits
30804558	LINC00461	Autism spectrum disorder
30804558	NEGR1	Autism and major depressive disorder (MTAG)
30804558	SORCS3	Autism and major depressive disorder (MTAG)
28540026	TCF4	Autism spectrum disorder or schizophrenia
28540026	TCF4	Autism spectrum disorder or schizophrenia
23453885	RNA5SP459 - TCF4	Five mental traits
23453885	TCF4	Five mental traits
31835028	SOX5	Eight mental traits
31835028	LINC00461	Eight mental traits
31835028	DCC	Eight mental traits
31835028	TCF4	Eight mental traits
31835028	SORCS3	Eight mental traits
31835028	LINC00461	Eight mental traits
31835028	SORCS3	Eight mental traits
31835028	SOX5	Eight mental traits
31835028	SYNE2, ESR2	Eight mental traits
31835028	TCF4	Eight mental traits
18839057	ASTN2, AL133284.1	Attention deficit hyperactivity disorder
26184070	SOX5	Oppositional defiant disorder dimensions in attention-deficit hyperactivity disorder
29325848	SOX5	Attention deficit hyperactivity disorder
29325848	MEF2C	Attention deficit hyperactivity disorder
30610198	LINC00461	Attention deficit hyperactivity disorder or cannabis use
30818988	LINC00461	Attention deficit hyperactivity disorder or caudate nucleus volume (pleiotropy)
32279069	ASTN2	Attention deficit hyperactivity disorder (persistent)
30478444	SORCS3	Attention deficit hyperactivity disorder
30478444	LINC00461	Attention deficit hyperactivity disorder

23453885	RNA5SP459 - TCF4	Five mental traits
23453885	TCF4	Five mental traits
31835028	SOX5	Eight mental traits
31835028	LINC00461	Eight mental traits
31835028	DCC	Eight mental traits
31835028	TCF4	Eight mental traits
31835028	SORCS3	Eight mental traits
31835028	LINC00461	Eight mental traits
31835028	SORCS3	Eight mental traits
31835028	SOX5	Eight mental traits
31835028	SYNE2, ESR2	Eight mental traits
31835028	TCF4	Eight mental traits
19571811	TCF4	Schizophrenia
26198764	TCF4	Schizophrenia
26198764	NEGR1	Schizophrenia
26198764	DCC	Schizophrenia
26198764	SORCS3	Schizophrenia
26198764	AC087235.1 - SOX5	Schizophrenia
26198764	SOX5	Schizophrenia
26198764	TCF4	Schizophrenia
31268507	TCF4	Schizophrenia
30285260	DCC	Schizophrenia
30285260	MEF2C	Schizophrenia
30285260	TCF4	Schizophrenia
30285260	MEF2C	Schizophrenia
30285260	DCC	Schizophrenia
30285260	TCF4	Schizophrenia
23894747	TCF4	Schizophrenia
30626913	RNA5SP459 - TCF4	Broad depression or schizophrenia
28991256	DCC	Schizophrenia
31740837	TCF4	Schizophrenia
31740837	TCF4	Schizophrenia
19571808	TCF4	Schizophrenia
31374203	DCC	Cognitive ability, years of educational attainment or schizophrenia (pleiotropy)
31374203	TCF4	Cognitive ability, years of educational attainment or schizophrenia (pleiotropy)
31374203	NEGR1	Cognitive ability, years of educational attainment or schizophrenia (pleiotropy)
31374203	LINC00461	Cognitive ability, years of educational attainment or schizophrenia (pleiotropy)
31374203	SORCS3	Cognitive ability, years of educational attainment or schizophrenia (pleiotropy)
31374203	ESR2	Cognitive ability, years of educational attainment or schizophrenia (pleiotropy)
29483656	AC087235.1 - SOX5	Schizophrenia
29121268	LINC00461	Bipolar disorder lithium response (categorical) or schizophrenia
28540026	TCF4	Autism spectrum disorder or schizophrenia
28540026	TCF4	Autism spectrum disorder or schizophrenia
21926974	TCF4	Schizophrenia
25056061	TCF4	Schizophrenia

23453885	RNA5SP459 - TCF4	Five mental traits
23453885	TCF4	Five mental traits
31835028	SOX5	Eight mental traits
31835028	LINC00461	Eight mental traits
31835028	DCC	Eight mental traits
31835028	TCF4	Eight mental traits
31835028	SORCS3	Eight mental traits
31835028	LINC00461	Eight mental traits
31835028	SORCS3	Eight mental traits
31835028	SOX5	Eight mental traits
31835028	SYNE2, ESR2	Eight mental traits
31835028	TCF4	Eight mental traits
30804565	TCF4	Insomnia
30804565	DCC	Insomnia
30804565	NEGR1	Insomnia
30804565	NEGR1	Insomnia
30804565	DCC	Insomnia
30804565	TCF4	Insomnia
30804566	RNA5SP459 - TCF4	Insomnia symptoms (never/rarely vs. sometimes/usually)
30804566	RNA5SP459 - TCF4	Insomnia symptoms (never/rarely vs. usually)
29728651	AC114971.1 - TMEM161B	Major depressive disorder
27479909	SORCS3	Major depressive disorder
29317604	THSD7A - TMEM106B	Plasma kynurenine levels in major depressive disorder
29942085	TCF4	Depression
29942085	DCC	Depression
29942085	LINC00461	Depression
29942085	DCC	Depression
29942085	TCF4	Depressed affect
29942085	DCC	Depressed affect
29942085	DCC	Depressed affect
29942085	AC022031.2, TCF4	Depressed affect
29942085	TCF4	Depressed affect
29942085	TCF4	Depressed affect
29942085	TCF4	Depressed affect
29942085	TCF4	Depressed affect
29942085	SORCS3	Depressed affect
29942085	LINC00461	Depressed affect
29942085	TMEM106B	Depressed affect
29942085	SORCS3	Depressed affect
29942085	DCC	Depressed affect
29942085	DCC	Depression
29942085	DCC	Depression
29942085	NEGR1	Depressed affect
29942085	SORCS3	Depression

29942085	TCF4	Depression
29942085	DCC	Depression
29942085	TMEM106B	Depression
29942085	DCC	Depression
31969693	THSD7A - TMEM106B	Major depressive disorder
29942085	THSD7A - TMEM106B	Depressed affect
29942085	TCF4	Depressed affect
29942085	TCF4	Depressed affect
29942085	TCF4	Depressed affect
29942085	DCC	Depressed affect
29942085	DCC	Depressed affect
29942085	TCF4, TCF4-AS1	Depressed affect
29942085	DCC	Depressed affect
29942085	SOX5	Depression
29942085	DCC	Depression
29942085	TCF4	Depression
29942085	DCC	Depression
32231276	SORCS3	Help-seeking from a GP
32231276	THSD7A - TMEM106B	Help-seeking from a GP
32231276	SORCS3	Help-seeking from a GP
29662059	RNA5SP459 - TCF4	Depression (broad)
29662059	TCF4	Depression (broad)
29662059	TMEM106B	Major depressive disorder (probable)
29662059	THSD7A - TMEM106B	Depression (broad)
29662059	SORCS3	Depression (broad)
29662059	SORCS3	Depression (broad)
29662059	DCC	Depression (broad)
29662059	TCF4	Depression (broad)
30626913	RNA5SP459 - TCF4	Broad depression or schizophrenia
31926635	DCC	Bipolar disorder or major depressive disorder
31926635	ASTN2	Bipolar disorder or major depressive disorder
31926635	CACNA1E	Bipolar disorder or major depressive disorder
31926635	SORCS3	Bipolar disorder or major depressive disorder
31926635	SYNE2, ESR2	Bipolar disorder or major depressive disorder
31926635	TCF4	Bipolar disorder or major depressive disorder
31926635	SOX5	Bipolar disorder or major depressive disorder
31926635	TMEM106B	Bipolar disorder or major depressive disorder
30718901	CACNA1E	Depression
30718901	TMEM161B	Depression
30718901	TCF4	Depression
30718901	SOX5	Depression
30718901	SORCS3	Depression
30718901	DCC	Depression
30718901	ESR2	Depression
30718901	TMEM106B	Depression

30718901	ASTN2	Depression
30804558	NEGR1	Autism and major depressive disorder (MTAG)
30804558	SORCS3	Autism and major depressive disorder (MTAG)
27089181	SORCS3	Depression
27089181	DCC	Depression
29700475	TMEM106B	Depression
29700475	DCC	Depression
29700475	TCF4	Depression
29700475	ASTN2	Depression
29700475	SOX5	Depression
29700475	SYNE2, ESR2	Depression
29700475	SORCS3	Depression
23453885	RNA5SP459 - TCF4	Five mental traits
23453885	TCF4	Five mental traits
31835028	SOX5	Eight mental traits
31835028	LINC00461	Eight mental traits
31835028	DCC	Eight mental traits
31835028	TCF4	Eight mental traits
31835028	SORCS3	Eight mental traits
31835028	LINC00461	Eight mental traits
31835028	SORCS3	Eight mental traits
31835028	SOX5	Eight mental traits
31835028	SYNE2, ESR2	Eight mental traits
31835028	TCF4	Eight mental traits
31043756	ASTN2	Bipolar I disorder
31754094	NEGR1	Bipolar disorder or body mass index
31926635	DCC	Bipolar disorder or major depressive disorder
31926635	ASTN2	Bipolar disorder or major depressive disorder
31926635	CACNA1E	Bipolar disorder or major depressive disorder
31926635	SORCS3	Bipolar disorder or major depressive disorder
31926635	SYNE2, ESR2	Bipolar disorder or major depressive disorder
31926635	TCF4	Bipolar disorder or major depressive disorder
31926635	SOX5	Bipolar disorder or major depressive disorder
31926635	TMEM106B	Bipolar disorder or major depressive disorder
29121268	LINC00461	Bipolar disorder lithium response (categorical) or schizophrenia
23453885	RNA5SP459 - TCF4	Five mental traits
23453885	TCF4	Five mental traits
31835028	SOX5	Eight mental traits
31835028	LINC00461	Eight mental traits
31835028	DCC	Eight mental traits
31835028	TCF4	Eight mental traits
31835028	SORCS3	Eight mental traits
31835028	LINC00461	Eight mental traits
31835028	SORCS3	Eight mental traits
31835028	SOX5	Eight mental traits

31835028	SYNE2, ESR2	Eight mental traits
31835028	TCF4	Eight mental traits

Eight mental traits: anorexia nervosa, attention-deficit/hyperactivity disorder, autism spectrum disorder, bipolar disorder, major depression, obsessive-compulsive disorder, schizophrenia, or Tourette syndrome (pleiotropy)

Five mental traits: Autism spectrum disorder, attention deficit-hyperactivity disorder, bipolar disorder, major depressive disorder, and schizophrenia (combined)

7. Supplementary Figure 1. Quantile-quantile plots of the observed meta-analysis statistics versus the expected statistics for MDD and PTSD

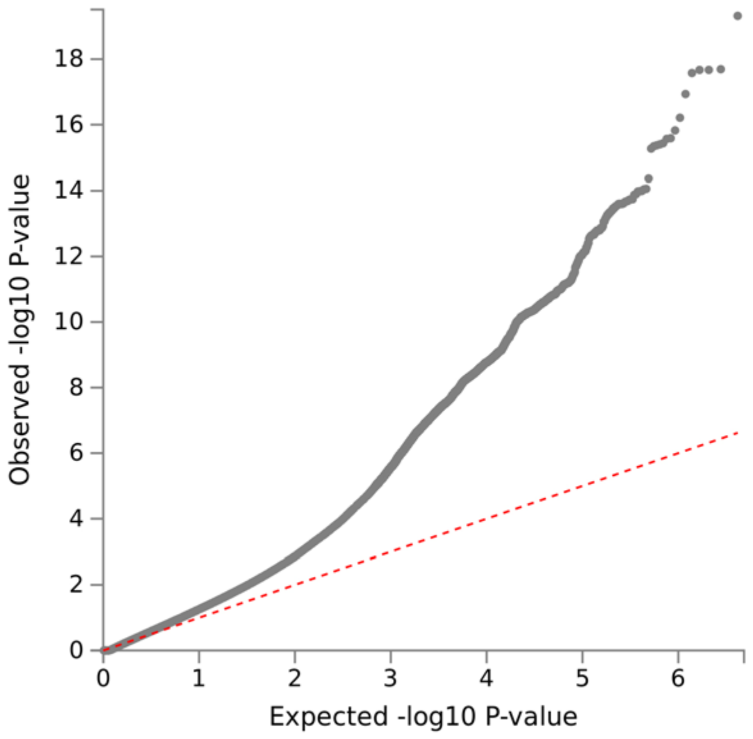

8. Supplementary Figure 2. Gene property analysis for tissue specificity in GTEx tissues

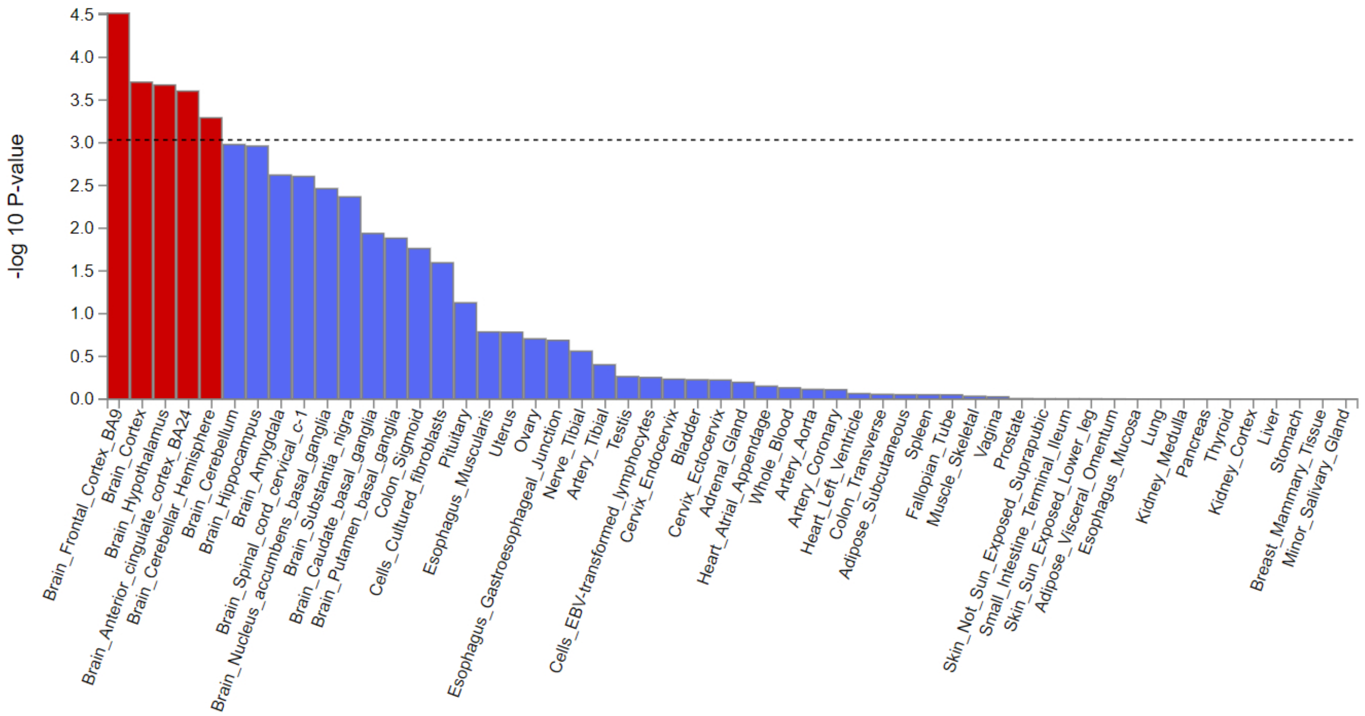

9. Supplementary Figure 3. Gene property analysis for tissue specificity in general GTEx tissues

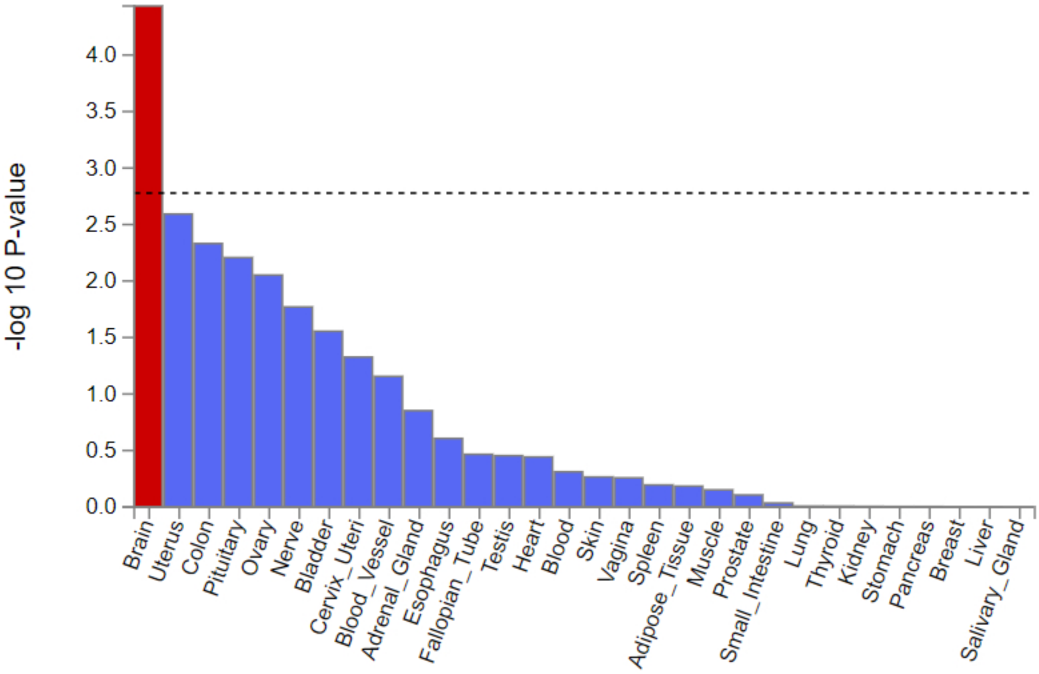

10. References

1. Howard DM, Adams MJ, Clarke TK, Hafferty JD, Gibson J, Shirali M, et al. Genome-wide meta-analysis of depression identifies 102 independent variants and highlights the importance of the prefrontal brain regions. *Nat Neurosci.* 2019;22(3):343-52.
2. Howard DM, Adams MJ, Shirali M, Clarke TK, Marioni RE, Davies G, et al. Genome-wide association study of depression phenotypes in UK Biobank identifies variants in excitatory synaptic pathways. *Nat Commun.* 2018;9(1):1470.
3. Hyde CL, Nagle MW, Tian C, Chen X, Paciga SA, Wendland JR, et al. Identification of 15 genetic loci associated with risk of major depression in individuals of European descent. *Nat Genet.* 2016;48(9):1031-6.
4. Wray NR, Ripke S, Mattheisen M, Trzaskowski M, Byrne EM, Abdellaoui A, et al. Genome-wide association analyses identify 44 risk variants and refine the genetic architecture of major depression. *Nat Genet.* 2018;50(5):668-81.
5. Allen NE, Sudlow C, Peakman T, Collins R, and Biobank UK. UK biobank data: come and get it. *Sci Transl Med.* 2014;6(224):224ed4.
6. Nagel M, Jansen PR, Stringer S, Watanabe K, de Leeuw CA, Bryois J, et al. Meta-analysis of genome-wide association studies for neuroticism in 449,484 individuals identifies novel genetic loci and pathways. *Nat Genet.* 2018;50(7):920-7.
7. Nievergelt CM, Maihofer AX, Klengel T, Atkinson EG, Chen CY, Choi KW, et al. International meta-analysis of PTSD genome-wide association studies identifies sex- and ancestry-specific genetic risk loci. *Nat Commun.* 2019;10(1):4558.
8. Genomes Project C, Auton A, Brooks LD, Durbin RM, Garrison EP, Kang HM, et al. A global reference for human genetic variation. *Nature.* 2015;526(7571):68-74.
9. Bulik-Sullivan B, Finucane HK, Anttila V, Gusev A, Day FR, Loh PR, et al. An atlas of genetic correlations across human diseases and traits. *Nat Genet.* 2015;47(11):1236-41.
10. Finucane HK, Bulik-Sullivan B, Gusev A, Trynka G, Reshef Y, Loh PR, et al. Partitioning heritability by functional annotation using genome-wide association summary statistics. *Nat Genet.* 2015;47(11):1228-35.
11. Bulik-Sullivan BK, Loh PR, Finucane HK, Ripke S, Yang J, Schizophrenia Working Group of the Psychiatric Genomics C, et al. LD Score regression distinguishes confounding from polygenicity in genome-wide association studies. *Nat Genet.* 2015;47(3):291-5.
12. Altshuler DM, Gibbs RA, Peltonen L, Dermitzakis E, Schaffner SF, Yu F, et al. Integrating common and rare genetic variation in diverse human populations. *Nature.* 2010;467(7311):52-8.
13. Frei O, Holland D, Smeland OB, Shadrin AA, Fan CC, Maeland S, et al. Bivariate causal mixture model quantifies polygenic overlap between complex traits beyond genetic correlation. *Nat Commun.* 2019;10(1):2417.
14. Zhu Z, Zhang F, Hu H, Bakshi A, Robinson MR, Powell JE, et al. Integration of summary data from GWAS and eQTL studies predicts complex trait gene targets. *Nat Genet.* 2016;48(5):481-7.
15. Watanabe K, Taskesen E, van Bochoven A, and Posthuma D. Functional mapping and annotation of genetic associations with FUMA. *Nat Commun.* 2017;8(1):1826.
16. de Leeuw CA, Mooij JM, Heskes T, and Posthuma D. MAGMA: generalized gene-set analysis of GWAS data. *PLoS Comput Biol.* 2015;11(4):e1004219.

17. Zhu Z, Zheng Z, Zhang F, Wu Y, Trzaskowski M, Maier R, et al. Causal associations between risk factors and common diseases inferred from GWAS summary data. *Nat Commun.* 2018;9(1):224.
